# The current landscape of therapeutic vaccination approaches for treatment of HPV-dependent malignancies

**DOI:** 10.1038/s41541-026-01426-8

**Published:** 2026-04-15

**Authors:** Johanne Audouze-Chaud, Ann-Katrin Schlosser, Angelika B. Riemer

**Affiliations:** 1 German Cancer Research Center (DKFZ) Heidelberg, Division of Immunotherapy & Immunoprevention, Heidelberg, Germany; 2 German Center for Infection Research (DZIF), partner site Heidelberg, Molecular Vaccine Design, Heidelberg, Germany; 3 Heidelberg University, Faculty of Biosciences, Heidelberg, Germany

**Keywords:** Cancer, Computational biology and bioinformatics, Immunology, Oncology

## Abstract

This review provides an overview of current therapeutic human papillomavirus (HPV) vaccination approaches. We review important criteria for their development, and present vaccines currently being actively followed in clinical trials, for precursor lesions or established cancers. Subsequently, we list approaches that reached clinical trial stage but were abandoned. Lastly, we discuss promising preclinical studies. We conclude with a brief overview of novel in silico tools developed to enhance vaccine design.

## Introduction

### Human papillomaviruses and cancer

Human papillomaviruses (HPV) comprise over 200 known double-stranded DNA viruses infecting basal keratinocytes of squamous epithelia of the skin and mucosae. Although most HPV types cause no disease or only benign skin and anogenital warts, at least 12 HPV types have been described as oncogenic and termed high-risk types (hrHPV). These include HPV16, 18, 31, 33, 35, 39, 45, 51, 52, 56, 58 and 59 (https://monographs.iarc.who.int/list-of-classifications). Of note, the low-risk types HPV6 and HPV11 are associated with the development of recurrent respiratory papillomatosis (RRP)^1^. In most cases, HPV infections, even high-risk infections, resolve spontaneously within two years, attesting for HPV susceptibility to immune attack. This has also recently been showcased by the first FDA approval of a therapeutic HPV vaccine, which targets the low-risk types HPV6 and HPV11 in the context of RRP (https://www.fda.gov/news-events/press-announcements/fda-approves-first-immunotherapy-recurrent-respiratory-papillomatosis). Persistent infections with high-risk HPV types are a necessary prerequisite for the development of multiple malignancies, including cervical, other anogenital as well as a subset of head and neck cancers (mainly oropharyngeal), overall accounting for 5% of all cancer cases worldwide, i.e. about 730,000 cases in 2022^2^. HPV16 is the most prevalent hrHPV and is responsible for ~60% of cervical cancers and over 85% of HPV-induced head and neck cancers^3,4^. HPV18 ranges second, causing ~15% of cervical cancer cases^3,5^. Before reaching the stage of advanced cancer, hrHPV are responsible for the development of precursor lesions, so-called intraepithelial neoplasia (e.g. CIN—cervical intraepithelial neoplasia), which are even more prevalent. As an example, in Germany, it is estimated that 6250 women are diagnosed with HPV-related cancers each year, while about 56,000 women are treated for precursor lesions within the same timeframe (https://www.rki.de/DE/Aktuelles/Publikationen/RKI-Ratgeber/Ratgeber/Ratgeber_HPV.html).

### Prevention, detection and treatment of HPV-related cancers

It is estimated that virtually all sexually active individuals will be exposed to HPV in the course of their life^6^. Prophylactic vaccines are based on viral-like particles (VLPs) consisting of the type-specific capsid protein L1 and protect individuals who have not yet been in contact with the targeted HPV types, from future infections with those HPV types, mainly by inducing neutralizing antibodies. Prophylactic HPV vaccines (e.g. Gardasil^TM^, Cervarix^TM^ or Cecolin^TM^) have been introduced in the national immunization program of 125 countries for girls, while 47 countries introduced them for boys as well^7,8^. However, only 28% of girls had received their first dose of HPV prophylactic vaccine in 2023 (https://www.who.int/data/gho/data/indicators/indicator-details/GHO/girls-aged-15-years-old-that-received-the-recommended-doses-of-hpv-vaccine), a number far below the objective of 90% global coverage to be reached by 2030, defined in the WHO strategy to accelerate the eradication of cervical cancer^7^. While effective in individuals naïve to HPV, prophylactic vaccines do not protect from the development of lesions in individuals that are already infected by hrHPV. Regular screenings with Papanicolaou (Pap) smears as well as HPV-DNA testing allow early detection of HPV infections in the cervical region. Similar screenings are performed in the anal region on high-risk individuals, such as HIV^+^ men having sex with men^9,10^. However, to date, there is no effective oropharyngeal cancer screening program available, due to the poor sensitivity of the available screening techniques for head and neck HPV infections^11^.

The current standard of care for detected lesions is dependent on their stage and location. Cervical precursor lesions are typically treated by conization or ablative treatments, which incur future childbearing risks. Other precursor lesions are typically ablated. When detected at an early stage, precursor lesions are not treated right away, but regularly monitored in the hope of a spontaneous regression, leading to strong psychological burden of the patients. Early cervical cancer is primarily treated by surgery or by radiotherapy with concurrent chemotherapy at later stages of the disease. Advanced cervical cancers are typically treated with platinum-based chemotherapy and radiotherapy^9^. The first line of treatment for oropharyngeal cancer consists of surgery and/or definitive radiotherapy at an early stage, while advanced stages are treated with platinum-based chemotherapy or resection followed by adjuvant radiotherapy^12^. Treatment options are the same regardless of HPV status, although HPV-positive head and neck cancers are associated with a better prognosis^13^. Therefore, de-escalation treatments for HPV-positive oropharyngeal carcinoma are currently being explored^14^. Immune checkpoint blockade has additionally been tested in HPV-positive cervical and oropharyngeal cancers, and, albeit limited efficacy, is now part of the treatment guidelines^15,16^.

The severe side effects associated with surgery, radiotherapy, and chemotherapy, together with the limited efficiency of immune checkpoint blockade, stress the need for novel treatment options. Therapeutic vaccination, representing a non-invasive approach with the promise of high specificity and low side effects, is an attractive alternative to existing treatments and is therefore actively researched. Unlike prophylactic vaccination, therapeutic vaccines aim at the induction of antigen-specific cytotoxic T lymphocytes (CTLs) that kill and thus eliminate infected or transformed target cells. As advanced HPV-induced lesions actively suppress local immunity, it would be especially interesting to introduce therapeutic HPV vaccination as a treatment for precursor lesions such as cervical intraepithelial neoplasia (CIN), vulvar or vaginal intraepithelial neoplasia (VIN, VaIN), penile intraepithelial neoplasia (PIN), or anal or peri-anal intraepithelial neoplasia (AIN, PAIN). Indeed, the WHO recently published preferred product characteristics (PPC) for therapeutic HPV vaccines targeting CIN^17^. It is worth noting that therapeutic HPV vaccination in this scenario can take advantage of a long therapeutic window, since an intraepithelial lesion generally takes over a decade (in average 15 years) to develop into cancer (https://www.who.int/news-room/fact-sheets/detail/human-papilloma-virus-and-cancer).

### Specific HPV proteins as targets for therapeutic HPV vaccines

As the vast majority of HPV-driven cancers arise from HPV16 and HPV18^3–5^, most HPV therapeutic vaccination approaches have focused on these two HPV types. In rare cases, other high-risk types were also included.

HPV encodes early (E) and late (L) genes. The proteins derived from the early genes (E1, E2, E4, E5, E6, and E7) are important for completing the viral life cycle, while the late gene products (L1, L2) are the structural proteins forming the viral capsid. All currently approved prophylactic vaccines target the L1 protein and induce virus-neutralizing antibodies. However, the late genes are not expressed in HPV-transformed cells and can therefore not represent targets for therapeutic vaccination^18^. This explains why prophylactic vaccines, which target the L1 protein, cannot exhibit any therapeutic effect.

E6 and E7, termed “oncoproteins”, maintain an active cell cycle in the host cells—a prerequisite for viral replication, and a feature that is typically no longer present in suprabasal keratinocytes. E7 binds to the cellular checkpoint pRb, breaking its interaction with the transcription factor E2F, which causes the transcription of several proteins, including cyclin E, cyclin A, and p16^INK4A^, and a subsequent premature entry of the cells into the S phase. Meanwhile, E6 binds the cellular ubiquitin ligase E6AP and the tumor suppressor molecule p53, promoting its rapid ubiquitination and degradation via proteasomes. This results in the prevention of cell cycle arrest and apoptosis, thereby allowing uncontrolled cell proliferation. Besides, E6 and E7 have been shown to mediate interferon signaling suppression^19^ and can alter c-myc expression, leading to further cell cycle dysregulation through abnormal cyclin and downstream transcription factor expression. Together, E6 and E7 induce abnormal and uncontrolled cell proliferation, contributing to the shift of the cell to a malignant state^20^. However, further genetic hits are required for malignant transformation, which are acquired over time in HPV-immortalized cells due to increasing genetic instability^21^.

As E6 and E7 expression is crucial for the viral life cycle, it is maintained throughout disease stages, from infection to advanced cancer^22^. No escape mutants can arise that would not express E6 or E7, as HPV-driven malignancies are dependent on their sustained expression^23^. Therefore, E6- and E7-derived epitopes are presented at the surface of all HPV-infected malignant cells, rendering them ideal targets for therapeutic vaccination^20^. Furthermore, viral proteins are immunologically foreign, therefore no central tolerance against them exists in the host, and immune responses elicited against them do not lead to potential autoimmunity. This allows a clear targeting of tumor cells in the context of therapeutic HPV vaccination.

Other early HPV proteins, such as E2 and E5, have also been considered as targets for therapeutic HPV vaccination^24^, but they are not expressed in all disease stages. For instance, E2 is typically lost upon viral genome integration^21^. E1-derived antigens were also found to be immunogenic in cervical cancer patients^25^, although those were discovered and explored more recently. Therefore, most therapeutic HPV vaccines use E6 and E7 as target antigens^26,27^. To achieve this, it is of utmost importance that these epitopes are actually being MHC-presented at the surface of HPV-infected/transformed cells and can be recognized by T cells^28^. An example of such a peptide is HPV16 E7_11-19_, which was detected in a conserved manner at the surface of HPV16-infected cells by mass spectrometry, induced specific T cell-mediated killing^29^, and was successfully translated to effective clinical immunotherapy in the context of a transgenic T cell receptor (TCR) T cell product^30^.

### Considerations for the development of HPV therapeutic vaccines

Therapeutic HPV vaccines have been developed on various platforms, which have been reviewed elsewhere^26,31,32^. Briefly, live-vector vaccines such as bacterial or viral vectors are highly immunogenic, but can be associated with higher toxicities and immune responses targeting the vector instead of the delivered antigen. DNA vaccines are easy to produce and well tolerated, but generally display less immunogenicity due to less efficient delivery approaches. RNA-based vaccines offer the advantage of being easily delivered to the cells, but special caution must be taken to avoid their degradation during manufacturing and transport. Peptide- and protein-based vaccines are stable and safe, and thus good antigen sources. However, they do not possess the inherent adjuvanticity of live-vector or nucleic acid-based vaccines, and therefore need to be combined with adjuvants. In the case of short peptide-based vaccines, they also require to be tailored to the patients’ HLA repertoire and be delivered in a way that ensures targeting to APCs. Finally, dendritic cell vaccines are highly immunogenic and stimulate both the innate and adaptive immune response. However, the manufacturing process is difficult and costly, making it challenging for higher scale purposes.

Another important consideration is the stage of cancer development at which a therapeutic vaccine intervention is being conducted. At the first stages of HPV infection (persistent infection, CIN1 in the cervix), the immune system is considered “ignorant”, as the virus employs mechanisms to evade innate immunity and delay the activation of an adaptive immune response^33,34^. These include, among others, low viral gene expression, as well as the alteration of protein functions and immune cell interactions^21,35,36^, and downregulation of MHCI and II expression^37,38^. At this stage, E2 is still expressed and represses E6 and E7 expression, and HPV remains mostly episomal. As the infection progresses, the tumor immune micro-environment switches from an ignorant to an immunocompromised state. As the HPV genome integrates into the host cell, the E2 gene is disrupted, allowing E6 and E7 to be overexpressed. The infected cells undergo immortalization, and an immunosuppressive environment settles: T_reg_s infiltrate the lesion site and proinflammatory cytokines and chemokines are repressed, while regulatory cytokines such as TGF-β are upregulated^33,34^. While specific T-cell mediated killing failed to be induced in the immune ignorance phase, the effector mechanisms of specific T cells are actively repressed in the immunocompromised state. For this reason, therapeutic vaccines administered alone should target precursor lesions (i.e. early stage intraepithelial neoplasia) to overcome immune ignorance. This approach, termed “cancer interception”, allows to eliminate the lesion before it develops into cancer, positioning itself between classical prophylaxis, or primary prevention, and treatment, or secondary prevention. If therapeutic vaccines are employed in advanced and thus immunocompromised cancer lesions, they need to be used as part of a combined treatment that also tackles the immunosuppressive milieu.

Therefore, we discriminated in this review between therapeutic vaccines targeting precursor lesions versus vaccines targeting cancers. We searched the relevant databases (PubMed, ClinicalTrials.gov) for therapeutic HPV vaccines tested in the clinic, to provide an up-to-date overview of approaches currently being pursued. Search terms included “HPV”, “therapeutic vaccine”, “therapeutic vaccination”, “therapeutic HPV vaccine”, “therapeutic HPV vaccination”. We additionally searched both databases with the names of the vaccines previously found to gain a complete overview of all the trials, including each vaccine. Additional information sources (conference reports, company press releases, and websites) were also included in the search. We included a section about therapeutic HPV vaccines that have reached the clinical stage but are not actively followed anymore. Next, we highlight a selection of promising vaccines tested at the preclinical stage, and give an outlook on in silico tools currently employed for vaccine design. Finally, we summarize recent advancements and discuss the major areas of improvement in the development of therapeutic HPV vaccines.

### Therapeutic HPV vaccines that are actively clinically investigated

Numerous vaccines have entered clinical trials in the last two decades. These include DNA, mRNA, whole protein, peptide, as well as viral and bacterial vector-based vaccines. An overview of currently clinically investigated vaccines, their target proteins and delivery systems, their developers, indication, and clinical trial phase reached is given in Table 1. While some vaccines have been tested in both precursor lesions and advanced cancers, others were assessed for only one disease state. As the lesion stage is an important parameter for therapeutic vaccine indication, we separated vaccines tested in precursor lesions from the ones tested in advanced cancers.

**Table 1:** Therapeutic HPV vaccines actively followed in clinical trials for either precursor lesions or cancer, sorted by vaccine type and highest clinical phase reached

Vaccine name	Vaccine type	Developer	Vaccine composition		Indication	Highest clinical phase
			HPV type and protein/peptide targeted	Delivery system/adjuvanted by		
VGX-3100	DNA	Inovio Pharmaceuticals / ApolloBio	HPV16 E6/E7 & HPV18 E6/E7		Precursor lesions	III
INO-3112/MEDI0457	DNA	Inovio Pharmaceuticals	HPV16 E6/E7 & HPV18 E6/E7	INO-9012 (DNA vector encoding IL-12)	Cancer	II
pNGVL4a “family”	DNA	Johns Hopkins/Papivax	**pNGVL4a-Sig/E7(detox)/HSP70 & pNGVL4a-CRT/E7(detox):** HPV16 E7 **pNGVL4a-CRTE6E7L2 (PVX-4):** HPV16 E6/E7/L2**pBI-11 (PVX-8):** HPV16 E6/E7 & HPV18 E6/E7 + Hsp70	Hsp70 or calreticulin	Precursor lesions & cancer	II II
GX-188E	DNA	Genexine	HPV16 E6/E7 & HPV18 E6/E7	Flt3L + GX-I7 (long-acting IL-7)	Cancer	II
VB10.16	DNA	Nykode	HPV16 E6/E7	CCL3L1	Cancer	II
NWRD08	DNA	Newish Technology	HPV16 E6/E7 & HPV18 E6/E7	-	Precursor lesions	I
BNT113	mRNA	BioNTech	HPV16 E6/E7	Lipoplex particles	Cancer	II/III
RG002	mRNA	RinuaGene Biotech	HPV16/18 E2/E6/E7	Lipid nanoparticles	Precursor lesions	I/II
CD40HVac	Protein	EnnoDC	HPV16 E6/E7	Anti-CD40 mAb, poly-ICLC	Cancer	I/II
TA-CIN^a^	Protein	Johns Hopkins/Papivax	HPV16 E6/E7/L2	-	Cancer	I
PDS0101	Synthetic long peptides	PDS Biotech	6 HPV16 E6/E7 synthetic long peptides (SLPs)	Cationic lipid nanoparticles	Cancer	III
ISA101/101b	Synthetic long peptides	ISA Pharmaceuticals (currently ISAbella Pharma)	13 (for ISA101) or 12 (for ISA101b) HPV16 E6/E7 overlapping SLPs: E6_1-32_, E6_19-50_, E6_41-65_, E6_55-80_, E6_71-95_, E6_85-109_, E6_91-122_, E6_109-140_, E6_127-158,_ E7_1-35_, E7_22-56_, E7_43-77_ (only in ISA101), E7_64-98_	Montanide	Cancer	II
PepCan	Synthetic long peptides	Univ. of Arkansas	4 HPV16 E6 SLPs: E6_1-45_, E6_46-80_, E6_81-115_, E6_116-158_	Candida albicans skin test reagent	Cancer	I/II
CUE-101	Short peptide	Cue Biopharma	HPV16 E7_11-20_, HLA-A2-restricted	Fusion protein of HLA-A2, HPV16 E7_11-20_, IL-2, and IgG1 Fc domain	Cancer	II
PRGN-2009	Viral vector	Precigen	35 non-HLA-restricted CTL epitopes of HPV16 E5/E6/E7 & HPV18 E6/E7	GC46 gorilla adenovirus	Cancer	II
Vvax001	Viral vector	ViciniVax	HPV16 E6/E7	Semliki Forest virus replicon	Precursor lesions	II
TA-HPV^a^	Viral vector	Johns Hopkins/Papivax	HPV16 E6/E7 & HPV18 E6/E7	Vaccinia virus	Precursor lesions & cancer	I II
TG4001	Viral vector	Transgene	HPV16 E6/E7	Modified Vaccinia Ankara virus (MVA); IL-2	Cancer	I/II
IGMKK16E7	Bacterial vector	Nihon Univ., Tokyo	HPV16 E7	*Lactobacillus paracasei*	Precursor lesions	I/II

*Hsp70* Heat shock protein 70, *Flt3L* Fms-related tyrosine kinase 3 ligand, *mAb* monoclonal antibody.

^a^Actively followed in clinical trials only in combination with pNGVL4a vaccines.

#### Vaccines against precursor lesions

Several vaccines have been tested in HPV-mediated precursor lesions (Table 2). These include DNA vaccines (VGX-3100, pNGVL4a family vaccines, NWRD08), one viral vector-based vaccine (Vvax001) and one bacterial vector-based vaccine (IGMKK16E7). Vaccines that have been tested in precursor lesions, but are currently only pursued in cancer, are listed in the “Advanced Cancers” section.

- DNA vaccines
  **VGX-3100**, targeting HPV16/18 E6/E7, has been tested in cervical, vulvar, anal, and peri-anal intraepithelial neoplasia grade 2/3 (CIN2/3, VIN2/3, AIN2/3, and PAIN2/3, respectively). In all trials, VGX-3100 was well tolerated, mainly leading to mild to moderate adverse events (AEs). Nevertheless, around 5–15% of patients across the different studies experienced grade ≥3 AEs, making VGX-3100 the least well tolerated HPV therapeutic vaccine. The vaccine, which was administered with electroporation, was first tested in a phase I trial on patients that had undergone surgery following CIN2/3 (NCT00685412^39^), where it was shown to be safe and gave indications of specific T cell induction. Several phase II trials were then completed, in which VGX-3100 showed promising results. In the HPV-003 trial (completed in 2014), 49.5% of vaccinated HPV16/18^+^ CIN2/3 patients experienced histopathological regression (versus 30.6% in the placebo group), and 40.2% reached HPV clearance (versus 14.3% in placebo group)^40,41^. Combination with local application of the TLR7 agonist imiquimod was shown to enhance the effect of the vaccine in the HPV-201 trial (completed in 2020)^42^, where HPV16/18^+^ VIN2/3 patients were treated with either the vaccine alone or the vaccine combined with imiquimod. At week 48, the combinatorial treatment led to absence of high-grade VIN and HPV16/18 clearance in 37.5% of patients, while this occurred in 15% of the patients that received the vaccine only. A phase II trial on AIN2/3 and PAIN2/3 (HPV-203) was completed in 2020 and showed a complete response of 9.1%^43^. VGX-3100 was also tested as monotherapy in two phase III studies as treatment for HPV16/18^+^ CIN2/3: the REVEAL1 (completed in 2020) and REVEAL2 trials (completed in 2022)^44,45^. In REVEAL1, 22.5% of the 138 vaccinated patients reached viral clearance and showed no evidence of CIN2/3 at week 36, against 11.1% of the 63 patients in the placebo group. At week 36 of REVEAL2, in the ITT (intention-to-treat) population, 27.6% of the treated patients exhibited no signs of CIN2/3 or HPV16/18 positivity anymore, vs. 8.7% in the placebo group. In a subgroup analysis of patients positive for an unspecified biomarker (*n* = 25), 28.6% of the treated patients exhibited no signs of CIN2/3 or HPV16/18 positivity, while this was not the case for any of the control patients. VGX-3100 is currently pursued by INOVIO’s Chinese partner, ApolloBio in another phase III trial.
  Vaccines from the **pNGVL4a family** include pNGVL4a-Sig/E7(detox)/HSP70, pNGVL4a-CRT/E7(detox) (both targeting HPV16 E7), PVX-4 (also called pNGVL4a-CRTE6E7L2, targeting HPVHPV16 E6/E7/L2) and PVX-8 (also called pBI-11, a construct targeting HPV16 E6/E7 and HPV18 E6/E7 and fused with Hsp70). They have been tested in low- and high-grade cervical precursor lesions, alone or in combination with imiquimod and other HPV vaccines such as vaccinia virus-based **TA-HPV** (targeting HPV16 E6/E7 and HPV18 E6/E7) and protein-based vaccine **TA-CIN** (targeting HPV16 L2/E6/E7), which had been proven to be safe on successfully treated HPV16^+^ cervical cancer patients (NCT02405221^46^). The combination with TA-CIN was named **PVX-2** (pNGVL4a-Sig/E7(detox)/HSP70 + TA-CIN) or **PVX-6** (PVX-4 + TA-CIN), while the combination of PVX-8 with TA-HPV is **PVX-7**. Phase I clinical trials demonstrated a good safety profile, no serious AEs being recorded in any of the trials. In a recent phase I trial (completed in 2023; NCT00788164) where the **pNGVL4a-Sig/E7(detox)/HSP70** vaccine was tested in combination with the viral vector-based vaccine TA-HPV and topical imiquimod, 41.7% of HPV16^+^ CIN3 patients displayed complete histological regression (all dose groups combined)^47^. In the highest dose group, this was achieved in 50% of the patients. **PVX-2**, the combination of the same vaccine with the protein-based vaccine TA-CIN, was well tolerated in HPV16^+^ low-grade cervical lesions patients in a phase II trial (PVX2TACIN17368)^48^. However, the study was terminated due to lack of funding. The NCT00988559 trial (completed in 2016), conducted in 39 HPV16^+^ CIN2/3 patients, combined **pNGVL4a-CRT/E7 (Detox)** with resection of the lesion at week 15, as well as local imiquimod application in some of the treatment groups^49^. At week 15 before the resection procedure, 16.7% to 33% of patients displayed an absence of CIN2/3 lesions. Three further trials, assessing PVX-4, PVX-6 and PVX-8, are currently recruiting (NCT04131413, NCT03913117) or not yet recruiting (NCT06210854).
  **NWRD08** targets HPV16/18 E6/E7 and is currently being tested on HPV16/18^+^ CIN2/3 in a non-randomized, open-label phase I trial (NCT06276101), for which no further information is available at the moment.
- mRNA vaccine
  **RG002** is an mRNA-based vaccine targeting HPV16/18 E2, E6, and E7. This vaccine is currently entering a phase I/II clinical trial for the treatment of HPV16/18^+^ CIN2/3 patients (NCT06273553, not yet recruiting).
- Viral and bacterial vector vaccines
  **Vvax001**, based on Semliki Forest virus, targets HPV16 E6/E7. It was first tested in a phase I study on patients with a history of CIN2/3 or cervical cancer (Vvax001-UMCG-01), where the vaccine was well tolerated and resulted in E6/E7-specific responses in IFNγ ELISpots in 83.3% of patients^50^. Results for a phase II study testing the effect of Vvax001 on HPV16^+^ CIN3 patients (Vvax001-UMCG-02) were recently released^51^. 94% of patients experienced an overall reduction in lesion size, while 50% exhibited histopathological regression to CIN1 or less (with 3/18 patients achieving complete regression). Additionally, 63% of patients displayed HPV16 clearance.
  **IGMKK16E7** vaccine contains *L. paracasei* bacteria harboring HPV16 E7 at the cell surface. The vaccine was tested at different doses in the phase I/II MILACLE study on HPV16^+^ CIN2/3 patients (completed in 2022)^52^. IGMKK16E7 was overall well tolerated (two grade 3 AEs were reported in the low dose group). At week 24, a dose-dependent proportion of the patients displayed complete regression to normal histology (30.2% in the highest dose group vs 12.5% of placebo-treated patients).

**Table 2:** Therapeutic HPV vaccines actively followed in clinical trials for treatment of precursor lesions

Vaccine	Trial phase	Indication	Combination treatment	Trial type	Trial status	Acronym/ Identifier	Outcome/ Efficacy	Ref.
VGX-3100	I	CIN2/3 post surgery	-	Non-randomized, open-label, no control group	Completed in 2011	HPV001 (NCT00685412)	18 patients (6 patients per dose group). No patients with grade ≥3 AEs. IFNγ ELISpot response rate (week 16): 78%. ELISA response rate (week 16): 67% (HPV16 E6), 94% (HPV16 E7), 39% (HPV18 E6), 100% (HPV18 E7).	^39^
	II	HPV16/18^+^ CIN2/3	-	Randomized, double-blind, placebo control	Completed in 2014	HPV-003 (NCT01304524)	107 treated patients vs 36 placebo. 4.8% of patients with grade ≥3 AEs (vaccine) vs 2.4% of patients with grade ≥3 AEs (placebo). Histopathological regression (week 36): 49.5% (vaccine) vs 30.6% (placebo). Complete response (week 36): 40.2% (vaccine) vs 16.7% (placebo).	^40,41^
	III	HPV16/18^+^ CIN2/3	-	Randomized, quadruple-blind, placebo control	Completed in 2020	HPV-301 / REVEAL 1 (NCT03185013)	138 treated patients vs 63 placebo. 9.6% of patients with grade ≥3 AEs (vaccine) vs 9.7% of patients with grade ≥3 AEs (placebo). Complete response (week 36): 22.5% (vaccine) vs 11.1% (placebo).	^44^
	II	HPV16/18^+^ VIN2/3	Imiquimod	Randomized, open-label, active control	Completed in 2020	HPV-201 (NCT03180684)	20 patients (vaccine) vs 8 patients (vaccine + imiquimod).^a^ 12% of patients with grade ≥3 AEs (vaccine) vs no patients with grade ≥3 AEs (placebo). Complete response (week 48): 15% (vaccine) vs 37.5% (vaccine + imiquimod).	^42^
	II	HPV16/18^+^ AIN2/3 or PAIN2/3	-	Open-label, no control group	Completed in 2020	HPV-203 (NCT03499795)	22 patients. 4.4% of patients with grade ≥3 AEs. Complete response (week 36): 9.1%.	^43^
	II	HIV^+^ HPV16/18^+^ AIN2/3 or PAIN2/3	-	Open-label, no control group	Completed in 2024	HPV-202 (NCT03603808)	N/A	-
	III	HPV16/18^+^ CIN2/3	-	Randomized, quadruple-blind, placebo control	Completed in 2022	HPV-303 / REVEAL 2 (NCT03721978)	21 treated patients vs 4 placebo.^a^ 14.3% of patients with grade ≥3 AEs (vaccine) vs no patients with grade ≥3 AEs (placebo). Complete response (week 36): 28.6% (vaccine) vs 0% (placebo).	^45^
pNGVL4a-Sig/E7 (detox)/ HSP70	I/II	HPV16^+^ CIN2/3	*-*	Non-randomized, open-label, no control group	Completed in 2010	JHOC-J0323 (NCT00121173)	15 patients (3–9 patients per dose group). No patients with grade ≥2 AEs. Complete response (week 15): 33% in the highest dose cohort.	^147^
pNGVL4a-Sig/E7 (detox)/ HSP70	I	HPV16^+^ CIN3	TA-HPV ( + Imiquimod)	Non-randomized, open-label, active control	Completed in 2023	J0656 (NCT00788164)	12 patients^a^ (3–6 patients per dose group). No patients with grade ≥2 AEs. Complete response (week 15): 41.7%^b^ (33–50%^c^).	^47^
pNGVL4a-CRT/E7 (detox)	I	HPV16^+^ CIN2/3	Resection ( + Imiquimod)	Non-randomized, open-label, no control group	Completed in 2016	J0866 (NCT00988559)	39 patients^a^ (6–9 patients per treatment group). No patients with grade ≥3 AEs. Complete response (week 15): 27.3%^b^ (16.7%-33%^c^).	^49^
pNGVL4a Sig/E7 (detox)/ HSP70	II	HPV16^+^ ASC-US, ASC-H or CIN1	TA-CIN (combination treatment “PVX-2”)	Open-label, no control group	Terminated (due to slow accrual/funding)	PVX2TACIN17368 (NCT03911076)	11 patients. No patients with grade ≥2 AEs.	^48^
pNGVL4a-CRTE6E7L2 (PVX-4)	I	HPV16^+^ CIN2/3	-	Non-randomized, open-label, no control group	Recruiting	J1955 (NCT04131413)	N/A	-
pNGVL4a-CRTE6E7L2 (PVX-4)	I	HPV16^+^ ASC-US or CIN1	(TA-CIN (combination treatment “PVX-6”))	Non-randomized, open-label, no control group	Recruiting	BB IND 18340 (NCT03913117)	N/A	-
pBI-11 (PVX-8)	II	HPV16/18^+^ ASC-US or CIN1	-	Randomized, triple-blind, placebo control	Not yet recruiting	CRMS-83553 (NCT06210854)	N/A	-
NWRD08	N/A	HPV16/18^+^ CIN1-3	-	Non-randomized, open-label, no control group	Unknown	NEWISH-HPV-001 (NCT05905354)	N/A	-
	I	HPV16/18^+^ CIN2/3	-	Open-label, no control group	Recruiting	NEWISH-HPV-101 (NCT06276101)	N/A	-
RG002	I/II	HPV16/18^+^ CIN2/3	-	Open-label, no control group	Not yet recruiting	NCT06273553	N/A	-
Vvax001	I	History of CIN2/3 or cervical cancer	-	Non-randomized, Open-label, no control group	Completed in 2017	Vvax001-UMCG-01 (NCT03141463)	12 patients (3 patients per dose group). No patients with grade ≥3 AEs. IFNγ ELISpot HPV16 E6/E7-specific response (week 7): 83.3%	^50^
	II	HPV16^+^ CIN3	-	Open-label, no control group	Unknown	Vvax001-UMCG-02 (NCT06015854)	18 patients. No patients with grade ≥3 AEs. Histopathological regression (week 25): 50%. Complete response (week 25): 16.7%^b^.	^51^
IGMKK16E7	I/II	HPV16^+^ CIN2/3	-	Randomized, double-blind, placebo control	Completed in 2022	MILACLE/ jRTC20311900	124 treated patients (39-43 patients per dose group) vs 40 placebo. 1.6%^b^ of patients with grade ≥3 AEs (vaccine) vs no patients with grade ≥3 AEs (placebo). Complete response (week 24): 12.2% (low vaccine dose) vs 15.0% (intermediate vaccine dose) vs 30.2% (high vaccine dose) vs 12.5% (placebo).	^52^

^a^Number of patients refers to the number of patients analyzed regarding vaccine efficacy in the study.

^b^Value was calculated by the authors using the available data.

^c^Depending on the dose group.

#### Vaccines against advanced cancers

Therapeutic vaccines were also tested in advanced cancer settings, albeit almost exclusively in combination with immune checkpoint blockade (ICB) or standard of care chemo- or radiotherapy (Table 3). Vaccines derived from the pNGVL4a construct and VGX-3100 (as INO-3112 contains VGX-3100) are the only ones currently still being tested for both precursor lesions and advanced cancers. Some vaccines were tested for precursor lesions, but were abandoned for this indication. These include GX-188E (NCT02139267), ISA101(b) (NL14015.000.06), VB10.16 (NCT02529930), TG4001 (NCT01022346) and PepCan (NCT02481414). However, they are still actively pursued in combination with other therapies in advanced cancer treatment (respective studies discussed below). Others, such as CD40HVac or BNT-113, have exclusively been tested in the cancer setting.

- DNA vaccines
  Two trials are currently ongoing with the **pNGVL4a-Hsp70** construct pBI-11 (PVX-8, targeting HVP16/18 E6/E7), combining it with the viral vector-based HPV16/18 E6/E7 vaccine TA-HPV (combination treatment PVX-7) in the context of hrHPV^+^ PD-L1^+^ recurrent/metastatic oropharyngeal squamous cell carcinoma (NCT05799144, phase II, recruiting^53^) and advanced cervical cancer (NCT06315257, phase I, recruiting).
  **GX-188E** is a DNA vaccine targeting HPV16 E6/E7 and HPV18 E6/E7, which was recently tested in a phase I/II trial in combination with pembrolizumab (anti-PD1) in HPV16/18^+^ recurrent/metastatic cervical cancer (GX-188E-005^54^). At week 24, 35% of the 60 patients showcased an overall response, including 8.3% complete responses. The combination treatment was more successful in PD-L1^+^ than PD-L1^−^ patients (38.9% vs 29.2% overall response rate). Two phase II trials are currently testing the combination of GX-188E with GX-I7 (a long-acting IL-7) and the anti-PD1 agents pembrolizumab (GENUINE^55^), or nivolumab (TRINITY), respectively. The GENUINE trial preliminary results showed major pathologic response in 63.6% of HPV16/18^+^ advanced, resectable head and neck squamous cell carcinoma and a complete response in 36.3% of patients.
  The DNA vaccine **VB10.16** targets HPV16 E6/E7, and its efficacy was assessed in a phase II trial in combination with atezolizumab (anti-PD-L1) in HPV16^+^ recurrent/metastatic cervical cancer patients (VB-C-02). An overall response rate of 19.1% was achieved after 1 year, including 6.4% complete responses^56^. Another phase I/II trial (VB-C-03) is currently recruiting to test VB10.16 in HPV16^+^ PD-L1^+^ recurrent/metastatic oropharyngeal cancer patients, in combination with pembrolizumab^57^, while a phase II trial (VB-C-04) in HPV16^+^ PD-L1^+^ recurrent/metastatic cervical cancer was withdrawn due to strategic reasons, as announced in a press release in August 2024 (https://nykode.com/wp-content/uploads/2024/08/240821_VB1016-strategic-repositioning-PR-FINAL.pdf).
  **INO-3112**, also called **MEDI0457**, another DNA vaccine, associates VGX-3100 (encoding HPV16/18 E6/E7, cf. precursor lesions) with another DNA vector (INO-9012) encoding for IL-12. It entered three clinical trials for HPV-driven head and neck cancers (NCT04001413, NCT0316224, NCT03439085), all combining the vaccine with the PD-L1 inhibitor durvalumab. NCT04001413 was withdrawn due to funding reasons. In the phase Ib/IIa trial (NCT03162224), completed in 2021, a disease control rate of 44.8% and an overall response rate of 27.6% was observed^58^. In another phase II trial (NCT03439085)^59^, similar results were achieved, with a disease control rate of 37% and an overall response rate of 21%. The company announced its intention to soon launch a phase III trial where INO-3112 will be combined with an anti-PD1 agent (toripalimab-tpzi) in HPV16/18^+^ patients with locally advanced OPSCC (https://ir.inovio.com/news-releases/news-releases-details/2025/INOVIO-Highlights-Anticipated-2025-Milestones-and-2024-Key-Accomplishments/default.aspx).
- mRNA vaccine
  **BNT113** is an mRNA vaccine specific for HPV16 E6/E7. In the phase I/II HARE-40 trial, completed in 2024, BNT113 was tested in 17 HPV16^+^ disease-free HNSCC patients (Arm 1A) and in 13 HPV16^+^ cancer patients (Arm 1B)^60^. Grade ≥3 adverse drug reactions were observed in 24% of patients, and specific immune responses were detected in both study arms. 71% of 7 patients in Arm 1B, that were evaluated with post-treatment tumor scans, achieved disease control. BNT113 is currently being further explored in combination with pembrolizumab in a phase II/III study on HPV16^+^ PD-L1^+^ recurrent/metastatic HNSCC (AHEAD-MERIT^61^). So far, 26.7% of patients experienced grade ≥3 AEs. The overall response rate was 40% (up to 48 months), and 4 out of 15 patients achieved complete response.
- Protein- and peptide-based vaccines
  CD40HVac is a protein-based vaccine, while PDS0101, ISA101(b), CUE-101, and PepCan are all peptide-based vaccines.
  **CD40HVac** contains a fusion of HPV16 E6/E7 proteins and an anti-CD40 monoclonal antibody. The HPVDCVax phase I/II study is currently recruiting HPV16^+^ OPSCC patients to test the vaccine, adjuvanted by poly-ICLC. So far, 12 patients have been recruited for the lowest dose cohort (HPVDCVax), and preliminary results of this cohort indicate a good safety profile of the vaccine^62^. A phase IIb trial for HPV16^+^ OPSCC patients is already planned (https://www.biospace.com/press-releases/ennodc-unveils-the-next-generation-of-dendritic-cell-targeting-immunotherapies-supported-by-new-leadership-and-an-evolved-strategy).
  **PDS0101**, a vaccine consisting of six HPV16 E6/E7 synthetic long peptides, encapsulated in cationic lipid nanoparticles, has been and is currently being tested in multiple phase I/II and phase II trials (20-C-0045, IMMUNOCERV, VERSATILE-002, MC200710). A first phase III trial (VERSATILE-003) is currently ongoing. In the VERSATILE-002 phase II study (completed in 2025), PDS0101 was administered to HPV16^+^ PD-L1^+^ recurrent/metastatic HNSCC patients in combination with pembrolizumab^63^. The vaccine was well tolerated, with 13% of patients experiencing grade ≥3 AEs. 34% of patients displayed an overall response, including 7.5% exhibiting a complete response, with a median overall survival of 30 months. A recent press release underlined encouraging results for the low PD-L1-expressing population (https://www.pdsbiotech.com/index.php/investors/news-center/press-releases/press-releases1/132-2025-news/1018-pds-biotech-sets-significant-benchmark-in-head-and-neck-canc2025-09-18-055003), which is especially hard to treat with conventional therapy. In the MC200710 phase I/II study, PDS0101 was also tested alone or in combination with pembrolizumab in patients with hrHPV^+^ advanced OPSCC^64^. While the overall response rate was 20% in the combination treatment group and 0% in the monotherapy group, high disease control rates of 70% and 100%, respectively, were observed. In the IMMUNOCERV study testing PDS0101 in combination with radiotherapy and cisplatin in advanced cervical cancer patients, 47% of patients experienced grade ≥3 AEs, 29% displayed over 90% gross tumor volume reduction at day 35 and 59% reached complete metabolic response by positron emission tomography computed tomography (PET CT)^65^.
  **ISA101** and **ISA101b**, vaccines consisting of HPV16 E6/E7 synthetic long peptides, completed several phase I/II and phase II trials. While the E6 SLPs contained in ISA101 and ISA101b are identical, ISA101b contains three E7 SLPs, while ISA101 contains four, in this way covering seven more amino acids (c.f. Table 1). In the ISA-HPV-01-12 phase I/II trial (completed in 2018), the response to ISA101b compared to ISA101 was assessed with different doses alone or in combination with chemotherapy and IFNα in a total of 72 HPV16^+^ recurrent/metastatic cervical cancer patients^66^. Overall, 27.8% of patients experienced grade ≥3 AEs. 43% of the patients displayed regression, while another 43% had stable disease. Of note, median overall survival of the patients that were vaccine responders was significantly higher than of non-responders (16.8 vs 11.2 months, respectively). In the NCT02426892 phase II study (completed in 2021), 24 HPV16^+^ incurable HNSCC or anogenital cancer patients were treated with ISA101 and nivolumab (anti-PD1). 8.3% of the 24 patients exhibited a complete response after 3 years, and the overall survival reached 12.5% in the same timeframe^67,68^. The vaccine is currently still being tested in HPV^+^ OPSCC patients in combination with pembrolizumab (anti-PD1) and cisplatin in the phase II HCC 19-082 study. Two additional phase II studies were recently published, where ISA101b was combined with the anti-PD1 agent cemiplimab in HPV16^+^ recurrent/metastatic oropharyngeal cancer (NCT03669718^69^), and cervical cancer (NCT04646005^70^), respectively. In the case of oropharyngeal cancer, the combination led to an overall response rate of 25.3% (versus 22.9% in the anti-PD1 monotherapy arm). In cervical cancer, the overall response rate reached 16.8%. However, a more detailed analysis of the cervical cancer trial revealed that the PD-L1^+^ patients were the ones benefitting the most from the combination (patients with at least 1% PD-L1 expression displayed a 24.1% response rate, while those with less than 1% reached 15.8% overall response rate).
  **CUE-101**, which is specific for HPV16 E7_11-20_, a short peptide restricted by HLA-A2, is currently being assessed in an active phase I trial on HPV16^+^ recurrent/metastatic HNSCC (CUE-101-01^71^). The vaccine was either tested as monotherapy or in combination with pembrolizumab (anti-PD1). 8% of the patients displayed grade ≥3 AEs during the dose escalation part of the trial, and no significant safety concern arose from the pembrolizumab combination. Among the 19 evaluable patients that received the combination of CUE-101 and pembrolizumab, 47% showed overall response, with two cases of complete response (https://cuebiopharma.gcs-web.com/news-releases/news-release-details/cue-biopharma-reports-new-complete-response-and-confirmed-50). The median progression-free survival (in the combination treatment group) reached 5.8 months. A phase II trial (NCT04852328) is currently recruiting HPV16^+^ OPSCC patients. In this study, CUE-101 is combined with standard of care treatment. Out of the 20 patients evaluable to date, only one patient experienced grade ≥3 AEs^72^.
  **PepCan** targets the HPV16 E6 protein, divided in four long peptides (cf. Table 1). This vaccine contains *Candida albicans* skin test reagent (Candin) as an adjuvant. Although in the context of precursor lesions, the Candin adjuvant has proven to be more efficient alone than the vaccine itself and is currently pursued as such^73^, PepCan is still actively followed in the context of advanced cancers. It was recently given to HNSCC patients in remission in a phase I/II trial (NCT03821272). There, 41.7% of the 12 vaccinated patients had experienced cancer recurrence 24 months post-treatment. Meanwhile, recurrence had occurred in 80% of the 5 placebo-treated patients at the same timepoint^74^.
- Viral vector vaccines
  TG4001 and PRGN-2009 are both based on viral vectors. **TG4001**, based on Modified Vaccinia Ankara virus, targets HPV16 E6/E7. In the TG4001.12 phase I/II study, TG4001 was tested in combination with the anti-PD-L1 agent avelumab in HPV16^+^ recurrent/metastatic anogenital cancer patients^75^. 2.3% of patients experienced serious AEs, none of them being related to the vaccine. Upon evaluation of 36 patients, an overall response rate of 22% was recorded, including one patient with complete response and seven patients with partial response. An update on the second part of the trial was recently published. There, 6.5% of the patients experienced serious treatment-related AEs in the combination treatment group, versus 4.5% in the avelumab monotherapy group. The overall response rate was 15.2% in the combination treatment group and 13.6% in the monotherapy group^76^. With this data, the study did not meet its primary objective of improvement (https://www.transgene.fr/wp-content/uploads/20241014_TG4001_Phase_II_update_EN.pdf). So far, no further updates on the future of this vaccine have been released.
  **PRGN-2009** uses GC46 gorilla adenovirus as a vector and targets 35 non-HLA restricted CTL epitopes of HPV16 E5/E6/E7 and HPV18 E6/E7. PRGN-2009 entered a phase I/II trial for the treatment of various HPV^+^ advanced/metastatic cancers, with or without the PD-L1- and TGFβ-targeting protein bintrafusp alfa^77^. 27% of patients in the latter group experienced grade ≥3 AEs, while none of the patients receiving the vaccine monotherapy experienced any. None of the 6 patients in the monotherapy arm showed any objective response, whereas the combination with bintrafusp alfa achieved a 20% objective response rate in 10 assessed patients with one complete response and one confirmed partial response. Three further trials are presently active (NCT05996523^78^) or recruiting for treatment of OPSCC and recurrent/metastatic cervical cancer (NCT06223568^79^, NCT06157151^80^).

**Table 3:** Therapeutic HPV vaccines actively followed in clinical trials for treatment of advanced cancer

Vaccine	Trial phase	Indication	Combination treatment	Trial type	Trial status	Acronym/ Identifier	Outcome/ Efficacy	Ref.
pNGVL4a-CRT/E7 (Detox)	I	HPV16^+^ HNSCC	Cyclophosphamide	Non-randomized open-label, no control group	Terminated (due to lack of funding)	J11129 (NCT01493154)	N/A	-
pBI-11 (PVX-8)	II	hrHPV^+^ PD-L1^+^ recurrent/ metastatic OPSCC	TA-HPV (combination treatment “PVX-7”) + Pembrolizumab (anti-PD1)	Open-label, no control group	Recruiting	VICCHN2208 (NCT05799144)	N/A	^53^
pBI-11 (PVX-8)	I	Advanced cervical cancer	TA-HPV (combination treatment “PVX-7”)	Randomized, open-label, no control group	Recruiting	J23105sIRB (NCT06315257)	N/A	-
GX-188E	I/II	HPV16/18^+^ recurrent/ metastatic cervical cancer	Pembrolizumab	Open-label, no control group	Completed in 2023	GX-188E-005 (NCT03444376)	60 patients^a^. 6.2% of patients with grade ≥3 AEs. Overall response rate (week 24): 35%. Complete response (week 24): 8.3%.	^54^
	II	HPV16/18^+^ advanced, resectable HNSCC	Pembrolizumab + GX-I7 (long-lasting IL-7) post surgery	Randomized, open-label, no control group	Recruiting	GENUINE (NCT05286060)	11 patients^a^ (in highest dose cohort). Major pathologic response^b^: 63.6%. Complete response: 36.3%.	^55^
	II	HPV16/18^+^ recurrent/ metastatic HNSCC	Nivolumab (anti-PD1) + GX-I7	Open-label, no control group	Recruiting	TRINITY (NCT05280457)	N/A	-
VB10.16	II	HPV16^+^ recurrent/ metastatic cervical cancer	Atezolizumab (anti-PD-L1)	Open-label, no control group	Completed in 2023	VB C-02 (NCT04405349)	47 patients^a^. 10% of patients with grade ≥3 AEs. Overall response rate (one year): 19.1%. Complete response as best overall response (one year): 6.4%.	^56^
	I/II	HPV16^+^ PD-L1^+^ recurrent/ metastatic OPSCC	Pembrolizumab	Non-randomized^c^, open-label, no control group	Recruiting	VB-C-03 (NCT06016920)	N/A	^57^
	II	HPV16^+^ PD-L1^+^ recurrent/ metastatic cervical cancer	Atezolizumab	Randomized, quadruple-blind, no control group	Withdrawn (due to company strategic reasons)	VB-C-04 (NCT06099418)	N/A	-
INO-3112	II	HPV16^+^ OPSCC	Durvalumab (anti-PD-L1)	Randomized, open-label, active control	Withdrawn (funding reasons)	NCT04001413	N/A	-
	Ib/IIa	HPV16/18^+^ recurrent/metastatic HNSCC	Durvalumab	Open-label, no control group	Completed in 2021	NCT03162224	29 patients^a^. 40% of patients with grade ≥3 AEs^d^. Disease control rate (week 16): 44.8%^d^ Overall response rate (45 months): 27.6%^d^	^58^
	II	HPV16/18^+^ recurrent/metastatic cancers	Durvalumab	Open-label, no control group	Terminated (project delayed)	NCT03439085	21 patients. 4.8% of patients with grade ≥3 AEs. Disease control rate (up to two years): 37%. Overall response rate (up to two years): 21%.	^59^
BNT113	I/II	HPV16^+^ recurrent HNSCC or anogenital cancer	-	Open-label, no control group	Completed in 2024	HARE-40 (NCT03418480)	17 patients with resected HPV16^+^ HNSCC, disease-free (Arm 1 A) and 13 patients with HPV16^+^ cancers (Arm 1B). 24% of patients with grade ≥3 adverse drug reactions Disease control rate^e^ (Arm 1B): 71%.	^60^
	II/III	HPV16^+^ PD-L1^+^ recurrent/ metastatic HNSCC	Pembrolizumab	Randomized, open-label, active control	Recruiting	AHEAD-MERIT (NCT04534205)	15 patients (in safety run in combination treatment group). 26.7%^d^ of patients with grade ≥3 AEs. Disease control rate (up to 48 months): 53% Overall response rate (up to 48 months): 40%. Complete response (up to 48 months): 26.7%^d^.	^61^
CD40HVac	I/II	HPV16^+^ OPSCC	-	Randomized, double-blind, placebo-controlled	Recruiting	HPVDCVax (NCT06007092)	12 patients (in lowest dose cohort). No patients with grade ≥3 AEs.	^62^
PDS0101	I/II	Various HPV^+^ advanced/ metastatic cancers	M7824 (anti-PD-L1/TGFβ fusion protein) + NHS-IL12	Open-label, no control group	Active	20-C-0045 (NCT04287868)	50 patients. 52% of patients with grade ≥3 AEs. Disease control rate: 34% Overall response rate: 22%. Complete response: 8%.	^148^
	II	Advanced cervical cancer	Radiation therapy + cisplatin	Open-label, no control group	Active	IMMUNOCERV (NCT04580771)	17 patients. 47% of patients with grade ≥3 AEs. Reduction of gross tumor volume >90% (5 weeks $\pm$ 5 days): 29%. Complete metabolic response (24 $\pm$ 2 weeks): 59%.	^65^
	II	HPV16^+^ PD-L1^+^ recurrent/ metastatic HNSCC	Pembrolizumab	Open-label, no control group	Completed in 2025	VERSATILE-002 (NCT04260126)	53 patients^d^. 13% of patients with grade ≥3 AEs. Overall response rate: 34%. Complete response: 7.5%^d^.	^63^
	I/II	hrHPV^+^ advanced OPSCC	Pembrolizumab	Randomized, single-blind, no control group	Active	MC200710 (NCT05232851)	10 patients (vaccine) vs 10 patients (vaccine + pembrolizumab). 5% of patients with grade ≥3 AEs. Disease control rate: 70%^d^ (vaccine) vs 100%^d^ (vaccine + pembrolizumab) Overall response rate: 0% (vaccine) vs 20% (vaccine + pembrolizumab) Complete response: 0% for both groups	^64^
	III	HPV16^+^ PD-L1^+^ recurrent/ metastatic HNSCC	Pembrolizumab	Randomized, open-label, active control	Active	VERSATILE-003 (NCT06790966)	N/A	-
ISA101/ ISA101b	I/II	HPV16^+^ recurrent/ metastatic cervical cancer	Carboplatin, paclitaxel, bevacizumab, IFNα	Non-randomized^c^, open-label, no control group	Completed in 2018	ISA-HPV-01-12 (NCT02128126)	72 patients (6–14 patients per treatment group). 27.8% of patients with grade ≥3 AEs. Disease control rate: 86%. Overall response rate: 43%. Complete response: 5.6%.	^66^
	II	HPV16^+^ incurable OPSCC	Utomilumab	Open-label, no control group	Terminated (due to slow accrual)	2017-0145 (NCT03258008)	N/A	-
	II	HPV16^+^ incurrable HNSCC or anogenital cancer	Nivolumab	Open-label, no control group	Completed in 2021	2014-1047 (NCT02426892)	24 patients. 8.3% grade ≥3 AEs. Overall response rate (4.5 years): 33.3%^d^. Complete response (4.5 years): 8.3%.	^67,68^
	II	HPV^+^ OPSCC	Pembrolizumab, cisplatin	Open-label, no control group	Active	HCC 19-082 (NCT04369937)	N/A	-
	II	HPV16^+^ recurrent/ metastatic oropharyngeal cancer	Cemiplimab (anti-PD1)	Randomized, triple-blind, active control	Active	NCT03669718	91 patients (vaccine + cemiplimab)^a^ vs 96 patients (cemiplimab only)^a^ 48% (vaccine + cemiplimab) vs 44.9% (cemiplimab only) of patients with grade ≥3 AEs. Overall response rate: 25.3% (vaccine + cemplimab) vs 22.9% (cemiplimab only).	^69^
	II	HPV16^+^ recurrent/ metastatic cervical cancer	Cemiplimab	Open-label, no control group	Completed in 2018	NCT04646005	113 patients. 30.1% of patients with grade ≥3 AEs. Overall response: 16.8%. Complete response as best overall response: 2.7%.	^70^
CUE-101	I	HPV16^+^ recurrent/ metastatic HNSCC	Pembrolizumab	Non-randomized, open-label, no control group	Active	CUE-101-01 (NCT03978689)	19 patients^a^ (vaccine) vs 19 patients^a^ (vaccine + pembrolizumab). 8% of patients with grade ≥3 AEs. Disease control rate: 37% (vaccine) vs 74% (vaccine + pembrolizumab) Overall response rate: 5.3%^d^ (vaccine) vs 47% (vaccine + pembrolizumab). Complete response: 0% (vaccine) vs 5.3%^d^ (vaccine + pembrolizumab).	^71^
	II	HPV16^+^ OPSCC	Standard of care	Non-randomized, open-label, no control group	Recruiting	NCT04852328	20 patients (10 patients per treatment group). 5% of patients with grade ≥3 AEs.	^72^
PepCan	I/II	Patients in remission of HNSCC	-	Randomized, double-blind, placebo control	Completed in 2025	217672 (NCT03821272)	12 patients (vaccine) vs 5 patients (placebo). Cancer recurrence rate (24 months): 41.7% (vaccine) vs 80% (placebo).	^74^
TG4001	I/II	HPV16^+^ recurrent/ metastatic anogenital cancer	Avelumab	Randomized, open-label, active control	Active	TG4001.12 (NCT03260023)	Phase Ib: 36 patients^a^ (vaccine + avelumab). 2.3% of patients with ≥ grade 3 AEs. Disease control rate: 42% Overall response rate: 22%. Complete response: 2.8%. Phase II: 46 patients (vaccine + avelumab) vs 44 patients (avelumab only). 6.5% (vaccine + avelumab) vs 4.5% (avelumab only) of patients with grade ≥3 AEs. Overall response rate: 15.2% (vaccine + avelumab) vs 13.6% (avelumab only).	^75,76^
PRGN-2009	I/II	Various HPV^+^ advanced/ metastatic cancers	Bintrafusp alpha	Non-randomized, open-label, no control group	Active	200104 (NCT04432597)	6 patients (vaccine) vs 10 patients^a^ (vaccine + bintrafusp alpha). no patients with grade ≥3 AEs (vaccine) vs 27% of patients with grade ≥3 AEs (vaccine + bintrafusp). Disease control rate: 67%^d^ (vaccine) vs 40%^d^ (vaccine + bintrafusp) Overall response rate: 0% (vaccine) vs 20% (vaccine + bintrafusp). Complete response: 0% (vaccine) vs 10% (vaccine + bintrafusp).	^77^
	II	HPV^+^ stage I-III OPSCC	Pembrolizumab	Open-label, no control group	Active	10001536 (NCT05996523)	N/A	^78^
	II	hrHPV^+^ stage I or II OPSCC	Docetaxel + cisplatin	Randomized, open-label, active control	Recruiting	10001730 (NCT06223568)	N/A	^79^
	II	HPV16/18^+^ PD-L1^+^ recurrent/ metastatic cervical cancer	Pembrolizumab	Randomized, open-label, active control	Recruiting	PRGN-2009-201 (NCT06157151)	N/A	^80^

^a^Number of patients refers to the number of patients analyzed regarding vaccine efficacy in the study.

^b^Defined as residual viable tumor of less than or equal to 10%.

^c^No randomization for dose escalation phase (phase 1), but randomization during dose expansion phase (phase 2).

^d^Value was calculated by the authors using the available data.

^e^Assessed in 7 patients by post-treatment tumor scans.

### Vaccine approaches that have reached the clinical stage but are no longer actively followed

Multiple therapeutic HPV vaccines have been tested in clinical trials, but are no longer actively followed for various reasons (Table 4).

- DNA vaccines
  The DNA vaccine **ZYC101a/Amolimogene** targeting HPV16 E6/E7 and HPV18 E6/E7 was tested until 2009 in a phase II/III trial on CIN2/3 patients (NCT00264732), although no results are available for this study. Another DNA vaccine was tested on high-grade VIN in a phase I/II clinical trial, namely an **HPV16 E6/E7 tattoo vaccine**^81^. Most of the AEs observed in this study were mild, although one patient presented a grade 3 AE. Out of five patients in the first cohort, two developed a complete response and one had a partial response. In the second cohort, which included nine patients, only three patients displayed a partial response, while the others did not experience any response. No further updates were released since 2021.
- Protein- and peptide-based vaccines
  Several protein vaccines have been tested in various HPV-driven lesions or tumors. **SGN-001101/HspE7** targets HPV16 E7 and uses Hsp65 as an adjuvant. In the NCT00052897 phase I/II trial on HIV^+^ AIN2/3 patients, most adverse events were mild to moderate, although dose-related toxicity was observed. However, after 48 weeks, only 26% of patients regressed to AIN1, while all others were non-responders^82^. In another phase II trial on CIN3 patients (NCT00075569), 22.5% of the 58 patients achieved a complete response, 55% had a partial response, and 19% had stable disease^83^. Of note, no differences were observed between women infected by HPV16 or other HPV strains, and it cannot be excluded that the results observed were due to spontaneous regression, as no control arm was included. **GTL001/ProCervix** targets both HPV16 and HPV18 E7 proteins, and uses a detoxified adenylate cyclase (CyaA) from *Bordetella pertussis* as an adjuvant. Two trials were started on HPV16/18^+^ patients with normal cytology or low-grade cervical lesion, a phase I (NCT02689726) and a phase II (NCT01957878) trial. The phase II trial included 47 patients. Adverse events occurred in all patients, among which 17 experienced severe grades. In the open-label portion of the study, 80% of the patients in the 600 µg vaccine/imiquimod combination group responded to the treatment. Similar responses were observed in 62.5% of patients in the placebo/imiquimod group and in 40% of patients in the 100 µg vaccine/imiquimod combination group^84^. The poor efficacy of this European study led to the termination of the phase I NCT02689726 trial in the US. **HPV16 E6E7 ISCOMATRIX** consists of a fusion protein of HPV16 E6/E7 and uses Iscomatrix (a component containing cholesterol, phospholipid and saponin^85^) as an adjuvant. It entered two phase I studies, one for HIV^+^ hrHPV^+^ AIN patients^86^ and one for CIN1/2/3 patients^87^. Although the vaccine was well tolerated, it showed limited success, with no AIN patients responding and only one CIN2/3 patient regressing to CIN1. **PD-E7**, another protein vaccine, targets HPV16 E7 and contains AS02B (*Haemophilius influenzae* protein D) as an adjuvant. In a phase I/II trial on HPV^+^ CIN1/3 patients, the subjects developed a vaccine-specific response. Nevertheless, the vaccine was not developed further^88^. Lastly, **L1E7-CVLP**, a protein vaccine targeting HPV16 E7 and L1, assembling into viral-like particles, was tested in a safety study^89^. However, no updates were released since 2007.
  **Hespecta** consists of two long E6 peptides, HPV16 E6_71-95_ and E6_127-158_ (also contained in the ISA101 vaccine), which are conjugated to a TLR2 ligand (Amplivant) as an adjuvant. This vaccine was tested in a phase I trial (NCT02821494) on 25 HPV16^+^ premalignant lesion patients distributed among 4 dose groups. Toxicity was limited to grade 1 or 2 and was dose-dependent. The T cell response measured was also shown to be dose-dependent, with measurable responses only in the highest dose group^90^. Subsequently, the vaccine was tested in a phase I trial in HPV16 + OSCC patients (NL-OMON41192), but further development was halted. The short peptide vaccine **CIGB-228** is specific for the HLA-A2-restricted peptide HPV16 E7_86-93_ and uses very small size proteoliposomes (VSSP) as an adjuvant and delivery platform. It was tested on HPV16^+^ CIN2/3 and presented a good safety profile (only grade 1 AEs were recorded). Three out of seven patients displayed complete regression and HPV clearance^91^. However, no further results regarding this vaccine were released since 2011. **DPX-E7**, which is specific for the HLA-A2-restricted epitope HPV16 E7_11-19_, was administered to HPV16^+^ recurrent/metastatic OPSCC, cervical or anal cancer patients, either alone or together with cyclophosphamide in a phase I/II study (NCT02865135^92^). Treatment was well tolerated in the 11 patients. In phase Ib, 16.7% of patients proved to be ELISpot responders, however, there were no responders among the 5 phase II patients. 33.3% phase I patients displayed stable disease. The same proportion was achieved in phase II for the combination therapy group, while all patients in the vaccine monotherapy group experienced disease progression. The vaccine is however, no longer pursued as the company was liquidated. Another peptide vaccine, **PCI-E7**, using a photochemical internalization technique (NCT02947854^93^) was tested in a phase I trial on healthy subjects. This vaccine contains two long E7 peptides, HPV16 E7_1-35_ & E7_62-98_. Vaccine doses equal or below 17.5 µg were well tolerated, while doses of 12.5 µg and above induced a specific immune response. However, this vaccine seems to be no longer followed as no update has been published since 2020.
- Cell-based vaccines
  Furthermore, many cell-based vaccines were tested. The **BVAC-C** vaccine is based on autologous B cells and monocytes loaded with α-galactosyl ceramide and transduced with HPV16 E6/E7 and HPV18 E6/E7. In the BVAC-C-P1 phase I/IIa trial (NCT02866006), although the vaccine was safe, only one out of 26 patients (3.8%) achieved complete response, while 4 patients (15.4%) exhibited partial response^94^. The subsequent trial for BVAC-C (phase II, NCT04800978) is classified as “unknown status”. Two HPV16/18 **E7-pulsed DC-based vaccines** were developed by the Universities of Arkansas, USA, and Jena, Germany, respectively, and tested in HPV16/18^+^ cervical cancer patients, though no objective response was obtained for either of the studies^95,96^. A variant of the E7-pulsed DC vaccine from the University of Arkansas, containing keyhole limpet hemocyanin (KLH) instead of IL-2, was tested in a phase I trial on cervical cancer patients, where it was found to be safe and immunogenic^97^. Another autologous monocyte-derived DC-based vaccine was developed by the National Taiwan University Hospital and tested in a phase I trial on HPV16^+^ cervical cancer (NCT00155766), for which the completion status remains unknown. The trials for **SQZ-eAPC-HPV** and **RTX-321** (both cell-based) were terminated, both because of corporate/sponsor decisions.
- Viral and bacterial vector vaccines
  The viral vector-based vaccine **AD/MG1-E6E7** was tested in a phase Ib trial (NCT03618953) which was terminated due to issues with the vaccine vector. A phase I/II clinical trial (HPV001) for **VTP-200** (an adenoviral vector-based vaccine specific for six early proteins of five hrHPV types), involving hr-HPV^+^ CIN1 patients, was recently completed, and an article is to be released soon (10.1093/cid/ciaf658). However, the Barinthus HPV program was abandoned. The **HB-200** vaccine (also called TheraT and Eseba-vec) uses two arenaviruses, namely lymphocytic choriomeningitis virus (LCMV) and pichinde virus (PICV), as vectors, and targets HPV16 E6/E7. HB-200 has been administered in a phase I/II trial in the context of HPV16^+^ recurrent/metastatic cancers together with the standard of care. Out of 42 patients, 14% experienced grade ≥3 AEs. The overall response rate among the 35 evaluable patients reached 43%, including 3 complete responses and 9 confirmed partial responses^98^. The developer, Hookipa Pharma, however, recently announced their intention to suspend their activities for financial reasons, and the HPV development program has so far not been taken up by another company. Due to this decision, another trial involving HB-200 for patients with recurrent/metastatic oropharyngeal cancer (NCT06513884) was withdrawn. A Modified Vaccinia Ankara virus-based approach, named MVA-E2, targeting bovine papillomavirus E2 protein, was also tested on CIN2/3^99^ and intraurethral flat condyloma^100^ patients. As this vaccine does not target human, but bovine papillomavirus, we chose not to include it as HPV vaccine in this review. Besides, no further mention of this approach has been published recently.
  Lastly, several vaccines based on bacterial vectors were tested in clinical trials, although their development was subsequently stopped. The *Listeria monocytogenes*-based vaccine **ADXS11-001**, targeting HPV16 E7, was tested in several trials on HPV16^+^ cervical cancer, CIN2/3, oropharyngeal cancer, and anal cancer patients. In most of these trials, patients experienced severe adverse events and dose-limiting toxicity. For instance, in the NCT01266460 phase II trial on cervical cancer patients, all of the 50 treated patients experienced AEs, among which 42% were grade 3 or 4. Only one patient had a complete response and two achieved partial response^101^. This vaccine also entered a phase III trial (NCT02853604) in cervical cancer, but the trial was terminated as none of the 110 patients survived the trial due to disease progression. The NCT01598792 phase I trial on oropharyngeal cancer patients was terminated because a patient suffered dose-limiting toxicity. Both **BLS_ILB_E710c** and **GLBL101c** target HPV16 E7 and are based on *Lactobacillus casei*. Both were tested in HPV16^+^ CIN3 patients. In the phase I/II trial for BLS_ILB_E710c, no grade 3 or higher AEs were recorded, and 75% of patients experienced clinical efficacy in the phase II part^102^. However, the absence of a control arm makes it difficult to discriminate the effect of the vaccine from spontaneous regression cases. GLBL101c was well tolerated, with no patients experiencing any serious AEs. 70% of the 10 patients treated with the optimal dose experienced a disease regression to CIN2 at week 9. 5 patients further regressed to LSIL or normal cytology 12 months after the vaccination. However, no follow-up studies were published since 2014^103^.

**Table 4:** Therapeutic HPV vaccines no longer pursued

Vaccine name	Vaccine type	Developer	Vaccine composition			Clinical trials	
			HPV type and protein/peptide targeted	Adjuvant/delivery system	Indication	Trial phase	Acronym/ Identifier/Ref.
ZYC101a/ Amolimogene	DNA	Eisai/MGI Pharma Biologics	HPV16 E6/E7 & HPV18 E6/E7	poly (D,L-lactide-co-glycolide) microparticles	CIN2/3	II/III	ZYC1-004 (NCT00264732)
HPV16 E6/E7 tattoo vaccine	DNA	Netherlands Cancer Institute	HPV16 E6/E7	Epitopes of HIV negative factor protein, P30 epitope from tetanus toxin, pan HLA-DR	HPV16^+^ VIN2/3	I/II	NTR4607^81^
SGN-00101/HspE7	Protein	Akela Pharma	HPV16 E7	Hsp65	RRP	II	SGN-00101-0005 (NCT00038714)
					HIV^+^ AIN2/3	I/II	NCI-2012-02507 (NCT00052897)^82^
					CIN2/3	II	NCI-3074 (NCT00060099)
					CIN3	II	NCI-5850 (NCT00075569)^83^
					HPV16^+^ CIN3	II	NCI-2012-02513 (NCT00054041)
					HPV16^+^ CIN1	II	NCI-2012-02623 (NCT00091130)
					CIN1-3	I	HspE7-00101-0601 (NCT00493545)
GTL001/ProCervix	Protein	Genticel	HPV16 E7 & HPV18 E7	CyaA	HPV16/18^+^ normal cytology and CIN1	I	PC10VAC05 (NCT02689726)
					HPV16/18^+^ normal cytology and CIN1	II	RHEIA-VAC (NCT01957878)^84^
HPV-16 E6E7 ISCOMATRIX	Protein	Univ. of Queensland	HPV16 E6/E7	ISCOMATRIX	hrHPV^+^ HIV^+^ AIN1-3	Ib	^86^
					CIN1-3	I	^87^
PD-E7	Protein	Univ. of Brussels	HPV16 E7	AS02B (*Haemophilius influenzae* protein D)	hrHPV^+^ CIN1 & HPV16^+^ CIN3	I/II	^88^
L1E7 CVLP	Protein	Univ. of Jena	HPV16 E7 & L1	Chimeric L1-based virus-like particles (CVLPs)	CIN2/3	I	^89^
Hespecta/ISA201	Synthetic long peptides	ISA Pharmaceuticals	HPV16 E6_71-95_ & E6_127-158_	Amplivant (TLR2 ligand)	HPV16^+^ (pre)malignant lesions	I	2014-000658-12 (NCT02821494)^90^
					Treated HPV16^+^ OSCC patients	I	NL-OMON41192
CIGB-228	Short peptide	CIGB	HPV16 E7_86-93_	VSSP	HPV16^+^ CIN2/3		^91^
DPX-E7	Short peptide	Dana-Farber/ Immunovaccine	HPV16 E7_11-19_, HLA-A2-restricted	Oil-based DepoVax (DPX) platform	HPV16^+^ recurrent/ metastatic OPSCC, cervical or anal cancer	I/II	15-578 (NCT02865135)^92^
PCI-E7	Synthetic long peptides	PCI Biotech	HPV16 E7_1-35_ & E7_62-98_	Fimaporfin; poly-ICLC	Healthy volunteers	I	NCT02947854^93^
BVAC-C	Cell-based	Cellid	HPV16 E6/E7 & HPV18 E6/E7	Autologous B cells and monocytes loaded with α-galactosyl ceramide	HPV16/18^+^ cervical cancer	II	ESR-18-14325 (NCT04800978)
					HPV16/18^+^ cervical cancer	I/II	BVAC-C-P1 (NCT02866006)^94^
E7-pulsed DCs	Cell-based	Univ. of Jena	HPV16 E7 & HPV18 E7	Autologous monocyte-derived DCs	HPV16/18^+^ cervical cancer		^95^
E7-pulsed DCs	Cell-based	Univ. of Arkansas	HPV16 E7 & HPV18 E7	Autologous monocyte-derived DCs; IL-2	HPV16/18^+^ cervical cancer		^96^
				Autologous monocyte-derived DCs; KLH	HPV16/18^+^ cervical cancer	I	^97^
DC vaccine	Cell-based	National Taiwan University Hospital	HPV16 E7 peptides	Autologous PBMC-derived DCs	HPV16^+^ cervical cancer	I	NCT00155766
SQZ-eAPC-HPV	Cell-based	SQZ Biotechnologies	HPV16 E6/E7	Autologous PBMCs treated with CD86-, IL-2- and IL-12-encoding mRNAs	HPV16^+^ solid tumors	I/II	COMMANDER-001 (NCT05357898)
RTX-321	Cell-based	Rubius Therapeutics	HPV16 E7-derived HLA-A*02:01-binding peptide	Engineered red blood cells expressing 4-1BBL and IL-12	HLA-A*02:01^+^ HPV16^+^ solid tumors	I	NCT04672980
AD/MG1-E6E7	Viral vector	Turnstone Biologics	HPV16 E6/E7 & HPV18 E6/E7	MG1 virus and Adenovirus	HPV^+^ solid tumors	I	Ad-MG1-E6E7-002 (NCT03618953)
VTP-200	Viral vector	Vaccitech/Barinthus	6 early proteins from 5 hrHPVs	Chimpanzee Adenovirus (ChAdOx) prime, MVA boost	hrHPV^+^ CIN1	I/II	HPV001 (NCT04607850)
HB-200 (HB-201 + HB-202)/Eseba-vec/ TheraT/	Viral vector	Hookipa Pharma	HPV16 E6/E7	Lymphocytic choriomeningitis virus (LCMV) and Pichinde virus (PICV)	HPV16^+^ recurrent/ metastatic cancer	I/II	H-200-001 (NCT04180215)^98^
					HPV16^+^ OPSCC	I/II	IRB21-1031 (NCT05108870)
					HPV16^+^ PD-L1^+^ recurrent/ metastatic OPSCC	II/III	H-200-004 (NCT06513884)
ADXS11-001/ Lovaxin C/ Lm-LLO-E7	Bacterial vector	Advaxis	HPV16 E7	*Listeria monocytogenes*; LLO	CIN2/3	II	Lm-LLO-E7-07 (NCT01116245)
					Cervical cancer	II	NCI-2011-02671 (NCT01266460)^101^
					HPV16^+^ OPSCC	I	2010-019916-20 (NCT01598792)
					Anal cancer	I/II	BrUOG 276 (NCT01671488)
					HPV16^+^ OPSCC	II	H-36001 GCO 13-1411 (NCT02002182)
					Cervical cancer	I	Lm-LLO-E7-1401 (NCT02164461)
					Cervical cancer or HPV16^+^ OPSCC	I/II	ADXS001-04 (NCT02291055)
					Cervical cancer	III	ADXS001-02 (NCT02853604)
					Anal cancer	II	ADXS001-06 (NCT02399813)
					HPV16^+^ CIN3	I/II	UMT2013-BLS-ILB-E710c (NCT02195089)^102^
BLS_ILB_E710c	Bacterial vector	BioLeaders	HPV16 E7	*Lactobacillus casei*	HPV16^+^ CIN3	I/IIa	^103^
GLBL101c	Bacterial vector	GENOLAC BL	HPV16 E7	*Lactobacillus casei*			

*Hsp65* Heat shock protein 65, *CyaA* detoxified adenylate cyclase from *Bordetella pertussis*, *VSSP* very small size proteoliposomes, *poly-ICLC* polyinosinic-polycytidylic acid stabilized with polylysine and carboxymethylcellulose, *KLH* Keyhole Limpet Hemocyanin, *LLO* Listeriolysin O.

### Preclinical studies

#### The importance of the choice of in vivo model

While the vaccines highlighted in the previous sections already entered the clinical trial phase, many more vaccines are currently in the preclinical pipeline. Part of the reason why observed preclinical efficacy fails to translate into the clinical phase is believed to be the in vivo setting in which these vaccines were preclinically tested. Early models included rabbit-, dog- and cattle-specific papillomaviruses, leading to papillomata in their respective host species^104^. The first rodent papillomavirus model was Mastomys natalensis papillomavirus (MnPV) in the multimammate mouse Mastomys coucha^105^. Murine papillomavirus (MmuPV1) was detected in 2011^106^ and subsequently used as a model for papillomavirus infection in several mouse strains^107^. Other rodent papillomaviruses were subsequently isolated^108^. These animal papillomavirus models were very useful to get insights into the biology of papillomavirus infection and cancer development, and optimal timing of prophylactic or therapeutic intervention^109^. However, human papillomavirus vaccines cannot be assessed in these animal papillomavirus models. To allow in vivo testing of therapeutic HPV vaccines, the HPV oncogenes were introduced into murine cells, serving both as immortalization factors as well as antigenic targets. In this setting, subcutaneous tumors were cured numerous times, yet their location and tumor microenvironment do not match those of a mucosal tumor, which is the natural site for all HPV-induced lesions. On the contrary, orthotopic models, i.e., in which the tumor develops at the correct anatomical site, are more likely to display a similar response as human patients. Further, subcutaneous and orthotopic tumors display distinct immune cell accessibility and dynamics, as was shown in a study comparing two models obtained by injecting the same murine HNSCC cell line either subcutaneously or orthotopically^110^. One of the most common orthotopic models used for HPV-driven cancers is the TC-1 cell line^111^, either applied intravaginally or injected into organs of the oral cavity^112,113^. TC-1 cells originated from lung cells of C57BL/6 mice and were transduced to express HPV16 E6 and E7, as well as a mutated hRas in order to be tumorigenic^111^. Luciferase was later added to make the cells detectable in vivo^112^. Another orthotopic model was developed to mirror oropharyngeal tumors, namely AT84-E7. To this end, oral squamous cell carcinoma AT-84 cells, derived from C3H mice, were genetically modified to stably express HPV16 E7 and luciferase^114^. Still, these models display only murine MHC molecules and are thus not suited to analyze HLA-restricted (i.e. human) immune responses. In particular, mice carrying the H2-D^b^ MHC molecule (C57BL/6 strain) strongly react to an immunodominant E7 epitope, namely E7_49-57_, which cannot bind most human HLA molecules^115^. The presence of one highly immunodominant epitope does not reflect the situation in the outbred human population. Therefore, results obtained in such murine models are not predictive for clinical success. Results from mouse strains that express human or humanized MHC molecules are more likely to translate into the clinic. Some vaccines from the pNGVL4a vaccine family were tested in HLA-A2 transgenic mice, in which the human HLA-A2 molecule was knocked-in in addition to murine MHCs^116^. This approach, however, still faces the challenge of the immunodominant E7 epitope being presented. More suitable are mouse strains such as A2.DR1, which are knocked-out for murine MHC and knocked-in for human HLA-DR1 and humanized MHCI (HLA-A2)^117^, or ABab-I mice, which were knocked-in for 7 HLA class I alleles^118^. Indeed, our group developed two in vivo models for the A2.DR1 mouse strain, first a syngeneic sarcoma-based model carrying the HPV16 oncogenes merely as antigenic targets^119^, and subsequently an HPV oncogene-dependent tumor model, mirroring the development of TC-1, named E6/7-lucA2^120,121^. The latter model is the first to combine HLA expression and orthotopic localization (intravaginal and oropharyngeal).

In the following section, we highlight selected preclinical studies that seem promising, in particular in regard to the in vivo model used or the robustness of the results obtained. We feature studies performed in orthotopic models, as well as in HLA-humanized mice, and using some novel and promising vaccine formulations (Table 5).

**Table 5:** Selection of promising preclinical vaccine candidates

Vaccine description		Model system description			Results	Ref.
HPV type and protein/peptide targeted	Vaccine composition	Mouse strain	Tumor cell line	Tumor location		
HPV16 E7	gDE7: mRNA encoding HPV16 E7 fused to HSV-1gD, encapsulated in LNPs	C57BL/6	HPV16 E6^+^/E7^+^ TC-1-luc	Vaginal & oral	Systemic HPV16-specific immune response and complete regression of ivag. and oral tumors.	^122^
HPV16 E6/E7 & HPV18 E6/E7	mHTV: mRNA encoding HPV16/18 E6/E7, encapsulated in LNPs	C57BL/6	HPV16 E6^+^/E7^+^ TC-1-luc	Vaginal	Limited tumor growth, leading to a better survival compared to PBS and an irrelevant mRNA vaccine and protective effects upon s.c. re-challenge. Increased IFNγ secretion by ELISpot in response to HPV16/18 E6/E7 challenge in non-human primates 4 weeks after immunization.	^123^
HPV16 E7_44-62_	HPV peptide and Q11 peptide connected by covalent bonding, self-assembling into nanofibers	C57BL/6	HPV16 E6^+^/E7^+^ TC-1-luc	Vaginal	Vaccination via s.c., i.n. and ivag. route induced systemic HPV16-specific immune response and ivag. tumor size reduction.	^124^
HPV16 E7 & HPV18 E7	hEDA-HPVE7-16/18: Fusion protein consisting of HPV16 E7, HPV18 E7 and human fibronectin EDA (TLR4 agonist) + poly ICLC	C57BL/6	HPV16 E6^+^/E7^+^ TC-1-luc	Vaginal	Vaccination induced systemic HPV16/18-specific immune response and suppression of ivag. tumor growth in 100% of mice.	^125^
HPV16 E7	αDEC205-E7: Anti-DEC205 monoclonal antibody fused to E7 + poly(I:C)	C57BL/6	HPV16 E6^+^/E7^+^ TC-1-luc	Vaginal & oral	Overall survival in vaginal setting (day 60): significantly higher in the fully treated group vs the poly-IC alone group and untreated group. Overall survival in oral setting (day 120): significantly higher in the fully treated group vs the poly-IC alone group.	^126^
HPV16 E7	Cationic nanosized hydrogel particle (cCHP nanogel) containing HPV16 E7, adjuvanted by cyclic di–adenosine monophosphate	C57BL/6	HPV16 E6^+^/E7^+^ TC-1-luc	Vaginal	Complete inhibition of tumor growth in the vaccinated group, which was significantly higher than the unimmunized and empty nanogel groups. Significantly increased IFNγ secretion by ELISpot in response to HPV16 E7 challenge in non-human primates 11 weeks after immunization.	^127^
HPV16 E7_11-19_	Silica nanoparticles loaded with HPV16 E7_11-19_ and poly(I:C)	A2.DR1	HPV16 E6^+^/E7^+^ PAP-A2	s.c.	Complete tumor regression in 5/9 mice, which was significantly higher than for mice treated with PBS or empty SiNPs with poly-IC.	^128^
HPV16 E6/E7 & HPV18 E6/E7	P016-2: N1-methylpseudouridine-containing mRNA encoding HPV16/18 E6/E7, encapsulated in LNPs	C57BL/6	HPV16 E6^+^/E7^+^ TC-1 & HPV18 E6^+^/E7^+^ MC38	s.c.	Significantly improved animal survival, especially when combined with anti-PD1 checkpoint inhibitor.	^129^
HPV16 E7	mRNA encoding HPV16 E7 encapsulated in LNPs	C57BL/6	HPV16 E6^+^/E7^+^ TC-1	s.c.	Significantly improved animal survival and reduced tumor growth compared to LNP control and induced strong specific cytotoxic response.	^130^
HPV16 E7_49-57_	LNP(E7Ag-E10/IMDQ-E10): LNPs containing E7_49-57_ peptide linked to an anionic glutamic acid decamer (E_10_) and imidazoquinoline	C57BL/6	HPV16 E6^+^/E7^+^ TC-1	s.c.	100% efficacy at day 20 in both prophylactic and therapeutic settings.	^131^
HPV16 E6/E7	T22H07: Oncolytic herpes simplex virus 1 (oHSV-1) expressing E6/E7 fusion protein M22H04: LNP-enveloped mRNA expressing tHA-mE7-mE6	C57BL/6	HPV16 E6^+^/E7^+^ TC-1	s.c.	Complete regression (day 35): 80% for combination treatment, max. 40% for single treatments. The combination of both vaccines induced significantly more specific CD8^+^ T cells than either vaccine alone.	^132^

*LNP* Lipid nanoparticles.

#### Studies in orthotopic models

Ramos da Silva and colleagues studied the effect of their mRNA-based lipid nanoparticle (LNP) vaccine (**gDE7**) in TC-1 orthotopic intravaginal and sublingual tumors^122^. The vaccine consisted of an mRNA encoding for HPV16 E7 fused with the herpes simplex virus type I glycoprotein D. Several versions of the vaccine were tested (non-replicating or self-amplifying mRNA, with or without nucleoside modification), reaching complete tumor regression with two vaccine formulations in both orthotopic settings. Lee et al. also tested an mRNA-based vaccine in the orthotopic setting. There, the HPV16/18 E6/E7-targeted vaccine (**mHTV**) was tested in an intravaginal TC-1 tumor model^123^. mHTV was able to limit the tumor growth in mice, leading to a better survival compared to PBS and an irrelevant mRNA vaccine. Further, mHTV exhibited protective effects in mice re-challenged with TC-1 subcutaneously. Finally, the immunogenicity of mHTV was also tested and validated in non-human primates, whose PBMCs exhibited increased IFNγ secretion 4 weeks after immunization by ELISpot in response to HPV16/18 E6/E7 challenge. A similar vaccine based on this platform is currently entering the clinical trial phase for the treatment of precursor lesions, with the name **RG002**.

Several studies tested promising peptide- or protein-based vaccines in orthotopic settings. The long peptide **HPV16 E7**_**44-62**_ was fused to Q11, a self-assembling peptide, and administered both subcutaneously and locally in the mucosa in genital TC-1 tumors^124^. The intravaginal route was most effective in limiting tumor growth and most potent in eliciting immune responses. Another study combined HPV16 and HPV18 E7 and the TLR4 agonist human fibronectin extra domain A (EDA) in a fusion protein, administered together with poly-ICLC, a derivative of the TLR3 agonist poly-IC^125^. This vaccine, named **hEDA-HPVE7-16/18**, was tested on TC-1 genital tumors, where it induced a significant IFNγ response after 7 days. A boost vaccine was administered 7 days after the first vaccination, leading to an overall survival of 100% at day 68, while the unvaccinated group exhibited 20% survival. An additional study led by Silva et al. investigated the effect of **αDEC205-E7 mAb** on vaginal and oral TC-1 tumors^126^. The vaccine, composed of an anti-DEC205 monoclonal antibody (targeting a DC C-type lectin receptor) fused to HPV16 E7, was associated with poly-IC. The treatment resulted in an overall survival of 80% in the vaginal setting at day 60, while the poly-IC alone group reached 40% survival and the non-treated mice had to be sacrificed by day 30. For the tongue tumors, the vaccine showed 100% efficacy, which was not significantly different from the poly-IC alone treatment group (75% survival). However, the vaccine allowed mice to survive to a tumor re-challenge, which was not the case for poly-IC alone. Finally, a recently published study developed a cationic nanosized hydrogel particle containing the E7 protein (**cCHP-nanogel-E7**) and adjuvanted with cyclic di-adenosine monophosphate. The vaccine was tested in intravaginal TC-1-luc tumors and showed a complete inhibition of tumor growth in comparison with unimmunized mice and mice immunized only with the nanogel vector. Additionally, non-human primates were immunized with the vaccine and developed a strong specific T cell response shown by ELISpot and flow cytometry^127^.

#### Studies in subcutaneous models

While the orthotopic setting better reflects the tumor microenvironment of the patients and is therefore better suited for preclinical testing, several promising vaccines have been tested subcutaneously.

A silica nanoparticle (**SiNP**) peptide-based vaccine targeting HPV16 E7_11-19_ in association with poly-IC^128^ was tested against s.c. tumors in an MHC-humanized mouse model. This vaccine was tested in HLA-humanized A2.DR1 mice, where it induced a vaccine-specific CD8^+^ T cell response. It was further tested on subcutaneous PAP-A2 tumors, a syngeneic HPV16 E6/E7^+^ tumor model for A2.DR1. The vaccine led to complete tumor regression in 5 out of 9 mice, which was significantly higher than for mice treated with PBS or empty SiNPs with poly-IC.

A recent paper by Zhang et al. tested an mRNA construct targeting HPV16 and HPV18 E6 and E7 on subcutaneous tumors^129^. The vaccine, termed **P016-2**, was encapsulated in lipid nanoparticles (LNP) and additionally encoded the granulocyte-macrophage colony-stimulating factor (GM-CSF). It was tested on TC-1 (HPV16^+^) and MC38 (HPV18^+^) tumor models, where it significantly improved animal survival. Of note, the effect of the vaccine was strengthened by combination with an anti-PD-1 checkpoint inhibitor, allowing complete tumor control and reaching 100% survival at day 24 in the MC38 model (*vs*. 80% for the vaccine monotherapy). Peng et al. tested another mRNA construct, targeting **HPV16 E7**, which was also encapsulated in LNP^130^. This vaccine induced a potent CD8 T cell response as well as changes in the tumor microenvironment, such as a decrease in Ki67 expression, and was associated with an improved survival and decreased tumor weight.

Ye and colleagues developed LNPs containing the HPV16 E7_49-57_ peptide, linked to an anionic glutamic acid decamer (E_10_) and further including the TLR7/8 agonist imidazoquinoline^131^. The vaccine, termed **LNP(E7Ag-E**_**10**_**/IMDQ-E**_**10**_**)** demonstrated 100% efficacy at day 20 in both a prophylactic and a therapeutic setting against TC-1 tumors. Further, it was able to elicit a specific CD8 T cell response. Finally, other studies combined two vaccines. As an example, a study conducted by Zhang et al. used a combination of **T22H07**, an oncolytic herpes simplex virus engineered to express an HPV16 E6/E7 fusion protein, and **M22H04**, an LNP-enveloped mRNA composed of HPV16 E6 and E7 sequences fused with haemagglutinin protein^132^. The two vaccines synergized and led to 80% complete regression 35 days after s.c. TC-1 implantation, while either vaccine alone reached 40% complete regression or less. The combination of both vaccines additionally induced significantly more specific CD8^+^ T cells than either vaccine alone.

### The growing role of in silico analyses in vaccine design

Recent developments in therapeutic vaccine design have highlighted the growing use of in silico tools to predict the safety and efficacy of future vaccines. Also in the HPV therapeutic vaccine field, many studies have started to adopt an in silico workflow for vaccine design^133–136^. This workflow typically starts by the identification of cytotoxic T lymphocyte (CTL) epitopes using the Immune Epitope Database (IEDB) and the NetMHCpan tool. Sometimes, additionally, helper T lymphocyte and B lymphocyte epitopes are also identified using IEDB, NetMHCIIpan and ABCpred or Bepipred. The antigenicity of the epitopes (and of the vaccine construct) can be predicted by using tools such as VaxiJen, and their ability to induce a cytokine response with servers such as IFNepitope, IL10pred or IL4pred. IEDB also offers tools to estimate the population coverage of the epitopes. Further, the allergenicity of the epitopes (and vaccine construct) can be assessed with servers such as AllergenFP or AllerTOP, while the toxicity can be predicted with the ToxinPred server. The physiochemical properties of the vaccine can be determined via the ProtParam tool. To predict efficient processing of the vaccine, an analysis with TAPPred can be run, in silico analyzing the vaccine affinity towards TAP molecules. In a next step, the secondary and tertiary structures of the vaccine can be determined using among others PSIPRED (secondary structure) and AlphaFold (tertiary structure). The most recent version of AlphaFold, namely AlphaFold3, was released recently^137^. The tertiary structure often requires to be visualized by another software (such as PyMOL) and refined (for instance using Galaxy-Refine). Subsequently, other parameters can be predicted, such as the vaccine affinity for TLRs (using ClusPro), its molecular dynamics (for instance with iMOLs) and immunogenicity (using C-ImmSim). A more complete review of the different tools available was recently published by Najafi et al.^138^. The advent of these machine learning/artificial intelligence (AI) tools allows to address previous common challenges in vaccine design, by processing and integrating bigger datasets, while accelerating the process. However, one must keep in mind that these analyses remain predictions, and thus need to be interpreted with caution and verified experimentally. For instance, immunogenicity assays are required to verify that the selected epitopes are indeed potent in inducing an immune response, and the different immune cells predicted to be triggered by the vaccine need to be experimentally verified as well. Furthermore, AI tools still need to be improved in terms of interpretability, transparency, reliability and robustness. The two latter aspects highly depend on the heterogeneity and availability of training data, another factor remaining to be harmonized^139^. With the rapid advances in AI technology, it is to be expected that in silico analyses will play an increasing role in the vaccine development pipeline.

## Discussion

In the rapidly evolving field of tumor immunotherapy, therapeutic cancer vaccines constitute a promising avenue due to their specificity and low side effects. However, therapeutic cancer vaccines often require to first identify tumor-specific or tumor-associated antigens. HPV therapeutic vaccines do not present this challenge, as all tumor cells have by definition been infected by HPV and present immunologically foreign HPV epitopes at their cell surface that are not expressed on healthy cells. For this reason, HPV therapeutic vaccination has been intensively researched, with some vaccines already reaching phase III trials, and continues to evolve today.

As therapeutic options currently already exist for both precursor lesions and HPV-driven cancers, it is important that therapeutic vaccines (including combination treatments) reach at least the same efficacy as the current standard of care for them to be approved. Furthermore, in particular at the precursor lesion stage, spontaneous regression is not a rare phenomenon. Besides, as every clinical trial design is different, it can be very difficult to compare one study to another. Therefore, clinical efficacy trials should include control arms to know whether the effect observed in the treatment arm could be due to spontaneous regression, and if the efficacy could reach that of a standard of care treatment. It has also been observed that some control arms behaved very differently between the phase II and phase III trials of a same vaccine, further stressing the need for including a control arm at each step of the development process. A reason for this is that multiple factors, such as age of the patient or HPV type, can influence the response to treatment and/or the spontaneous regression of a lesion. For this reason, it is necessary that the control arm is as closely matched as possible to the treated group when assessing a new HPV therapeutic vaccine.

Because all trials are designed differently (control arm inclusion, HPV-type restriction, type of lesion/cancer treated, combination with other treatment modalities, etc.), it is extremely difficult to compare studies. Furthermore, meta-analyses are hampered by the fact that not all the studies and trials are recorded on clinicaltrials.gov or other databases, making it extremely difficult to get a complete overview of all the ongoing research. Besides, even if the studies are recorded, the results are not always added to the database, strengthening this effect even more. Nonetheless, two meta-analyses of HPV therapeutic vaccine clinical trials have been performed^140,141^. Some of the approaches included in the meta-analyses are no longer actively followed and thus are listed in Table 4. We also only focused on vaccines targeting actual HPV-derived proteins, and therefore did not include vaccines such as MVA-E2, targeting bovine papillomavirus, or UCPVax, using Tert as a target protein.

As an example for a meta-analysis, Alouini and Pichon published a systematic review^141^, where 843 records were identified, among which only 9 randomized controlled trials (RCTs) met the criteria to qualify for qualitative synthesis. 7 of these RCTs (677 participants) involved CIN2/3 and demonstrated lesion regression after treatment. However, 2 of these had a high-risk for bias and one did not include statistical analysis. As a result, four trials (598 participants) were analyzed^40,52,142,143^. Even so, a pooling of the p-values revealed a significant difference between vaccine and placebo groups. The 2 other RCTs focused on advanced cervical cancer, where the vaccine treatment did not show a significant difference in outcome^144,145^. Of note, these two studies were performed in the era before checkpoint blockade combination therapy.

These results suggest that therapeutic HPV vaccination as a monotherapy would be more efficient at the stage of precursor lesions. This is corroborated by the biological mechanisms occurring at the different stages, with an immunosuppressing microenvironment being present already at the advanced precursor lesion stage (CIN3)^34^, while spontaneous regression can still occur in the case of precursor lesions. This also is in line with the WHO preferred product characteristics, which recommend to target precursor lesions^17^. However, many vaccine approaches that were unsuccessful in the precursor lesion setting are now being tried on advanced cancers, and some vaccines even were exclusively assessed in this setting. This can be explained by the fact that in the case of advanced diseases, therapeutic vaccines can be combined with other treatments, mainly immune checkpoint inhibitors (ICIs), and act synergistically. This is not possible in the intraepithelial neoplasia stage, as ICIs can have potentially lethal side effects. Secondly, this paradox can be explained by the difficulty to find investors to finance trials at the precursor lesion stage, since for this application, vaccines need to be better and as safe as the standard of care (typically consisting of conization or ablation). This is not the case for advanced cancers where the standard of care either has been already tried and failed, or will be associated with severe side effects in any case.

Despite those challenges, it is surprising that no breakthrough in the field of HPV therapeutic vaccination has been achieved to date, even in precursor lesions, given the multitude of approaches that have been tried. One possible explanation for this could be the use of unsuitable murine models. Indeed, subcutaneous models do not accurately reflect the tumor immune microenvironment found in oral or anogenital tumors, which is better mirrored by orthotopic models. As such, a vaccine that is promising in a subcutaneous setting could fail to elicit a response in an orthotopic one. Further, most studies are performed on C57BL/6 mice, which exhibit the immunodominant HPV epitope E7_49-57_. Therefore, vaccines eliciting immune responses against this epitope will obtain excellent results in these mice, but will not elicit any response in humans, where this epitope is not presented. For this reason, HLA-humanized mice represent a more accurate platform for testing new vaccine candidates. Additionally, the limited success encountered so far could be explained by the choice of vaccine vehicle, which might fail to elicit a cytotoxic T cell response. Another potential explanation for the limited success of therapeutic vaccines in precursor lesions could be that clinical trials on precursor lesions often include CIN3 patients, which, from an immune evasion point of view, resembles more the cancer stage. Studies on “true” precursor lesions, where the immune microenvironment is not yet immunosuppressive, should ideally be performed on infected healthy individuals or CIN1 patients. This implies that large studies would be needed to account for the amount of spontaneous regression.

Altogether, the efficacy and translatability of therapeutic HPV vaccine approaches could be improved by implementing the following points: vaccines should be tested in appropriate murine models (i.e. with a truly orthotopic tumor setting and a human MHC expression system); precursor lesions should be targeted to follow the principle of cancer interception; and finally, every trial should include a control group matching the vaccine-treated group as closely as possible to discriminate spontaneous regression and showcase the effect of the tested vaccine.

Recent advances have shown that biomarkers such as HPV methylation and p16/Ki67 dual stain positivity are predictors for an enhanced risk of disease progression. p16/Ki67 dual staining has now been FDA approved as a measure to sort positive patients for further examination^146^. This information could help in understanding the efficacy of new HPV therapeutic vaccines^34^. In particular, vaccination studies could be focused on these patients, and it would be useful to see if an increased portion of these patients are vaccine non-responders.

In conclusion, while HPV therapeutic vaccines seem to have encountered modest success so far, promising approaches are currently being tested in clinical trials. Therapeutic vaccine monotherapy should be indicated for precursor lesions rather than for advanced cancer stages, while combination therapies are a viable possibility in cancer. Further approaches are being developed and tested preclinically. New animal models are being developed to meet the need to assess vaccine efficacy in the most clinically representative setting possible. Finally, in silico tools are now being integrated in the vaccine design pipeline, with the aim of developing more efficacious vaccine platforms.

## Data Availability

No datasets were generated or analysed during the current study.

## References

[1] Ramos, M. L., Ueha, R., Goto, T., Matsumoto, N. & Kondo, K. Pathogenesis of recurrent respiratory papillomatosis and potential novel treatment strategies. *Auris Nasus Larynx* **52**, 381–387 (2025). 40516276 10.1016/j.anl.2025.05.011

[2] Bray, F. et al. Global cancer statistics 2022: GLOBOCAN estimates of incidence and mortality worldwide for 36 cancers in 185 countries. *CA Cancer J. Clin.* **74**, 229–263 (2024). 38572751 10.3322/caac.21834

[3] de Sanjose, S. et al. Human papillomavirus genotype attribution in invasive cervical cancer: a retrospective cross-sectional worldwide study. *Lancet Oncol* **11**, 1048–1056 (2010). 20952254 10.1016/S1470-2045(10)70230-8

[4] Lechner, M., Liu, J., Masterson, L. & Fenton, T. R. HPV-associated oropharyngeal cancer: epidemiology, molecular biology and clinical management. *Nat. Rev. Clin. Oncol.* **19**, 306–327 (2022). 35105976 10.1038/s41571-022-00603-7 PMC8805140

[5] Schiffman, M. et al. Carcinogenic human papillomavirus infection. *Nat. Rev. Dis. Prim.* **2**, 16086 (2016). 27905473 10.1038/nrdp.2016.86

[6] Chesson, H. W., Dunne, E. F., Hariri, S. & Markowitz, L. E. The estimated lifetime probability of acquiring human papillomavirus in the United States. *Sex. Transm. Dis.* **41**, 660–664 (2014). 25299412 10.1097/OLQ.0000000000000193 PMC6745688

[7] World Health Organization. *Global Strategy To Accelerate The Elimination Of Cervical Cancer As A Public Health Problem*. (2020).

[8] World Health Organization. Human papillomavirus vaccines: WHO position paper (2022 update). *Wkly. Epidemiol. Rec.* **50**, 645–672 (2022).

[9] Cohen, P. A., Jhingran, A., Oaknin, A. & Denny, L. Cervical cancer. *Lancet* **393**, 169–182 (2019). 30638582 10.1016/S0140-6736(18)32470-X

[10] Stier, E. A. et al. International Anal Neoplasia Society’s consensus guidelines for anal cancer screening. *Int. J. Cancer* **154**, 1694–1702 (2024). 38297406 10.1002/ijc.34850

[11] Berman, T. A. & Schiller, J. T. Human papillomavirus in cervical cancer and oropharyngeal cancer: One cause, two diseases. *Cancer* **123**, 2219–2229 (2017). 28346680 10.1002/cncr.30588

[12] Mody, M. D., Rocco, J. W., Yom, S. S., Haddad, R. I. & Saba, N. F. Head and neck cancer. *Lancet* **398**, 2289–2299 (2021). 34562395 10.1016/S0140-6736(21)01550-6

[13] Johnson, D. E. et al. Head and neck squamous cell carcinoma. *Nat. Rev. Dis. Prim.* **6**, 92 (2020). 33243986 10.1038/s41572-020-00224-3 PMC7944998

[14] Mehanna, H. et al. Radiotherapy plus cisplatin or cetuximab in low-risk human papillomavirus-positive oropharyngeal cancer (De-ESCALaTE HPV): an open-label randomised controlled phase 3 trial. *Lancet* **393**, 51–60 (2019). 30449623 10.1016/S0140-6736(18)32752-1 PMC6319250

[15] Monk, B. J. et al. Integration of immunotherapy into treatment of cervical cancer: Recent data and ongoing trials. *Cancer Treat. Rev.* **106**, 102385 (2022). 35413489 10.1016/j.ctrv.2022.102385 PMC10697630

[16] Huang, Y., Lan, Y., Zhang, Z., Xiao, X. & Huang, T. An update on the immunotherapy for oropharyngeal squamous cell carcinoma. *Front. Oncol.* **12**, 800315 (2022). 35372036 10.3389/fonc.2022.800315 PMC8965058

[17] World Health Organization. *WHO Preferred Product Characteristics For Therapeutic HPV Vaccines*. (2024).

[18] Yu, L., Majerciak, V. & Zheng, Z. M. HPV16 and HPV18 genome structure, expression, and post-transcriptional regulation. *Int. J. Mol. Sci.* **23**, 4943 (2022). 35563334 10.3390/ijms23094943 PMC9105396

[19] Lo Cigno, I., Calati, F., Albertini, S. & Gariglio, M. Subversion of host innate immunity by human papillomavirus oncoproteins. *Pathogens* **9**, 292 (2020). 32316236 10.3390/pathogens9040292 PMC7238203

[20] Pal, A. & Kundu, R. Human papillomavirus E6 and E7: the cervical cancer hallmarks and targets for therapy. *Front. Microbiol.* **10**, 3116 (2019). 32038557 10.3389/fmicb.2019.03116 PMC6985034

[21] McBride, A. A. & Warburton, A. The role of integration in oncogenic progression of HPV-associated cancers. *PLoS Pathog* **13**, e1006211 (2017). 28384274 10.1371/journal.ppat.1006211 PMC5383336

[22] Schichl, K. & Doorbar, J. Regulation and deregulation of viral gene expression during high-risk HPV infection. *Viruses* **17**, 937 (2025). 40733555 10.3390/v17070937 PMC12299310

[23] zur Hausen, H. Papillomaviruses and cancer: from basic studies to clinical application. *Nat. Rev. Cancer* **2**, 342–350 (2002). 12044010 10.1038/nrc798

[24] Eberhardt, C. S. et al. Functional HPV-specific PD-1(+) stem-like CD8 T cells in head and neck cancer. *Nature* **597**, 279–284 (2021). 34471285 10.1038/s41586-021-03862-z PMC10201342

[25] Peng, X. et al. Novel canonical and non-canonical viral antigens extend current targets for immunotherapy of HPV-driven cervical cancer. *iScience* **26**, 106101 (2023). 36876126 10.1016/j.isci.2023.106101 PMC9978627

[26] Chabeda, A. et al. Therapeutic vaccines for high-risk HPV-associated diseases. *Papillomavirus Res* **5**, 46–58 (2018). 29277575 10.1016/j.pvr.2017.12.006 PMC5887015

[27] Vonsky, M. S., Runov, A. L., Gordeychuk, I. V. & Isaguliants, M. G. Therapeutic vaccines against human papilloma viruses: achievements and prospects. *Biochemistry* **84**, 800–816 (2019). 31509730 10.1134/S0006297919070101

[28] Becker, J. P. & Riemer, A. B. The importance of being presented: target validation by immunopeptidomics for epitope-specific immunotherapies. *Front. Immunol.* **13**, 883989 (2022). 35464395 10.3389/fimmu.2022.883989 PMC9018990

[29] Riemer, A. B. et al. A conserved E7-derived cytotoxic T lymphocyte epitope expressed on human papillomavirus 16-transformed HLA-A2+ epithelial cancers. *J. Biol. Chem.* **285**, 29608–29622 (2010). 20615877 10.1074/jbc.M110.126722 PMC2937992

[30] Nagarsheth, N. B. et al. TCR-engineered T cells targeting E7 for patients with metastatic HPV-associated epithelial cancers. *Nat. Med.* **27**, 419–425 (2021). 33558725 10.1038/s41591-020-01225-1 PMC9620481

[31] Wang, R. et al. Human Papillomavirus vaccine against cervical cancer: opportunity and challenge. *Cancer Lett* **471**, 88–102 (2020). 31812696 10.1016/j.canlet.2019.11.039

[32] Zheng, Q. et al. Advancing the fight against cervical cancer: the promise of therapeutic HPV vaccines. *Vaccines* **13** (2025). 10.3390/vaccines13010092 PMC11768687 39852871

[33] Grabowska, A. K. & Riemer, A. B. The invisible enemy—how human papillomaviruses avoid recognition and clearance by the host immune system. *Open Virol. J.* **6**, 249–256 (2012). 23341860 10.2174/1874357901206010249 PMC3547646

[34] Dull, P. M. et al. Meeting report: considerations for trial design and endpoints in licensing therapeutic HPV16/18 vaccines to prevent cervical cancer. *Vaccine* **42**, 126100 (2024). 39004526 10.1016/j.vaccine.2024.07.001 PMC11413486

[35] Lau, L., Gray, E. E., Brunette, R. L. & Stetson, D. B. DNA tumor virus oncogenes antagonize the cGAS-STING DNA-sensing pathway. *Science* **350**, 568–571 (2015). 26405230 10.1126/science.aab3291 PMC12974531

[36] Moerman-Herzog, A. & Nakagawa, M. Early defensive mechanisms against human papillomavirus infection. *Clin. Vaccin. Immunol* **22**, 850–857 (2015). 10.1128/CVI.00223-15 PMC4519724 26063238

[37] Ashrafi, G. H., Haghshenas, M. R., Marchetti, B., O’Brien, P. M. & Campo, M. S. E5 protein of human papillomavirus type 16 selectively downregulates surface HLA class I. *Int. J. Cancer* **113**, 276–283 (2005). 15386416 10.1002/ijc.20558

[38] Westrich, J. A., Warren, C. J. & Pyeon, D. Evasion of host immune defenses by human papillomavirus. *Virus Res* **231**, 21–33 (2017). 27890631 10.1016/j.virusres.2016.11.023 PMC5325784

[39] Bagarazzi, M. L. et al. Immunotherapy against HPV16/18 generates potent TH1 and cytotoxic cellular immune responses. *Sci. Transl. Med.* **4**, 155ra138 (2012). 23052295 10.1126/scitranslmed.3004414 PMC4317299

[40] Trimble, C. L. et al. Safety, efficacy, and immunogenicity of VGX-3100, a therapeutic synthetic DNA vaccine targeting human papillomavirus 16 and 18 E6 and E7 proteins for cervical intraepithelial neoplasia 2/3: a randomised, double-blind, placebo-controlled phase 2b trial. *Lancet* **386**, 2078–2088 (2015). 26386540 10.1016/S0140-6736(15)00239-1 PMC4888059

[41] Bhuyan, P. K. et al. Durability of response to VGX-3100 treatment of HPV16/18 positive cervical HSIL. *Hum. Vaccin. Immunother.* **17**, 1288–1293 (2021). 33175656 10.1080/21645515.2020.1823778 PMC8078703

[42] Inovio Pharmaceuticals. *A Phase 2, Randomized, Open Label, Efficacy Study of VGX-3100 Delivered Intramuscularly Followed by Electroporation With CELLECTRA™ 2000 Alone or in Combination With Imiquimod, for the Treatment of HPV-16 and/or HPV-18 Related High Grade Squamous Intraepithelial Lesion (HSIL) of the Vulva*. (NCT03180684) [accessed on 09.12.2025]; Available from: https://clinicaltrials.gov/study/NCT03180684.

[43] Inovio Pharmaceuticals. *A Phase 2, Open Label, Study of VGX-3100 Delivered Intramuscularly (IM) Followed by Electroporation (EP) for the Treatment of HPV-16 and/or HPV-18 Related Anal or Anal/Peri-Anal, High Grade Squamous Intraepithelial Lesion (HSIL), (AIN2, AIN3, PAIN2, PAIN3) in Individuals That Are Seronegative for Human Immunodeficiency Virus (HIV)-1/2*. (NCT03499795) [accessed on 09.12.2025]; Available from: https://clinicaltrials.gov/study/NCT03499795.

[44] Inovio Pharmaceuticals. *A Prospective, Randomized, Double-Blind, Placebo-Controlled Phase 3* S*tudy of VGX-3100 Delivered Intramuscularly Followed by Electroporation With CELLECTRA™-5PSP for the Treatment of HPV-16 and/or HPV-18 Related High Grade Squamous Intraepithelial Lesion (HSIL) of the Cervix*. (NCT03185013) [accessed on 09.12.2025]; Available from: https://clinicaltrials.gov/study/NCT03185013.

[45] Inovio Pharmaceuticals. *A Prospective, Randomized, Double-Blind, Placebo-Controlled Phase 3 Study of VGX-3100 Delivered Intramuscularly Followed by Electroporation With CELLECTRA™ 5PSP for the Treatment of HPV-16 and/or HPV-18 Related High Grade Squamous Intraepithelial Lesion (HSIL) of the Cervix*. (NCT03721978) [accessed on 09.12.2025]; Available from: https://clinicaltrials.gov/study/NCT03721978.

[46] Gaillard, S. et al. Vaccination with an HPV therapeutic vaccine (TA-CIN) in patients with HPV16 associated cervical cancer yields robust immunologic responses: differential effects by vaccination site (1273). *Gynecol. Oncol.* **176**, S171–S172 (2023).

[47] Maldonado, L. et al. Intramuscular therapeutic vaccination targeting HPV16 induces T cell responses that localize in mucosal lesions. *Sci. Transl. Med.* **6**, 221ra213 (2014). 10.1126/scitranslmed.3007323 PMC4086631 24477000

[48] Einstein, M. H. et al. Safety run-in of intramuscular pNGVL4a-Sig/E7(detox)/HSP70 DNA and TA-CIN protein vaccination as treatment for HPV16+ ASC-US, ASC-H, or LSIL/CIN1. *Cancer Prev. Res.* **16**, 219–227 (2023). 10.1158/1940-6207.CAPR-22-0413 PMC10068439 36607735

[49] Sidney Kimmel Comprehensive Cancer Center at Johns Hopkins. *A Pilot Study of pnGVL4a-CRT/E7 (Detox) for the Treatment of Patients With HPV16+ Cervical Intraepithelial Neoplasia 2/3 (CIN2/3)*. (NCT00988559) [accessed on 09.12.2025]; Available from: https://clinicaltrials.gov/study/NCT00988559. 10.1016/j.ygyno.2015.11.026 PMC4724445 26616223

[50] Komdeur, F. L. et al. First-in-human phase I clinical trial of an SFV-based RNA replicon cancer vaccine against HPV-induced cancers. *Mol. Ther.* **29**, 611–625 (2021). 33160073 10.1016/j.ymthe.2020.11.002 PMC7854293

[51] Eerkens, A. L. et al. Vvax001, a therapeutic vaccine, for patients with HPV16-positive high-grade cervical intraepithelial neoplasia: a phase II trial. *Clin. Cancer Res.* **31**, 1016–1026 (2025). 39849926 10.1158/1078-0432.CCR-24-1662

[52] Kawana, K. et al. Phase I and II randomized clinical trial of an oral therapeutic vaccine targeting human papillomavirus for treatment of cervical intraepithelial neoplasia 2 and 3. *JNCI Cancer Spectr.* **7**, pkad101 (2023). 38001029 10.1093/jncics/pkad101 PMC10748578

[53] Gibson, M. K., Roden, R., Wu, T. C. & Heimann-Nichols, E. Phase II trial assessing safety, efficacy and immune correlates of heterologous prime-boost with pBI-11 (IM) and TA-HPV (IM) plus pembrolizumab for advanced, PD-L1 CPS≥1, hrHPV+ oropharyngeal cancer. *J. Clin. Oncol.* **42**, TPS6118–TPS6118 (2024).

[54] Lim, M. C. et al. GX-188E DNA vaccine plus pembrolizumab in HPV 16- and/or 18-positive recurrent or advance cervical cancer: a phase 2. *trial. eClinicalMedicine* **74**, 102716 (2024). 39823099 10.1016/j.eclinm.2024.102716 PMC11736335

[55] Kim, H. R. et al. Neoadjuvant pembrolizumab, GX-188E, and GX-I7 in patients with human papillomavirus-16- and/or 18-positive head and neck squamous cell carcinoma: Single-arm, phase 2 trial with single cell transcriptomic analysis and artificial intelligence-powered spatial analysis. *J. Clin. Oncol.* **41**, 6075–6075 (2023).

[56] Hillemanns, P. et al. Safety and efficacy of the therapeutic DNA-based vaccine VB10.16 in combination with atezolizumab in persistent, recurrent or metastatic HPV16-positive cervical cancer: a multicenter, single-arm phase 2a study. *JITC* **13** (2025). 10.1136/jitc-2024-010827 PMC11749841 39773564

[57] Khalique, S. et al. 941TiP A phase I/IIa, open-label, dose-finding trial to evaluate safety, immunogenicity, and anti-tumour activity of VB10.16 in combination with pembrolizumab in patients with unresectable recurrent or metastatic HPV16-positive head and neck squamous cell carcinoma (R/M HNSCC). *Ann. Oncol.* **35**, S653 (2024).

[58] MedImmune. *A Phase 1b/2a, Multi-Center Open-Label Study to Evaluate the Safety and Efficacy of Combination Treatment With MEDI0457 (INO-3112) and Durvalumab (MEDI4736) in Patients With Recurrent/Metastatic HPV Associated Head and Neck Squamous Cancer*. (NCT03162224) [accessed on 09.12.2025]; Available from: https://clinicaltrials.gov/study/NCT03162224.

[59] M. D. Anderson Cancer Center. *A Phase 2, Open-Label Study to Evaluate Efficacy of Combination Treatment With MEDI0457 (INO-3112) and Durvalumab (MEDI4736) in Patients With Recurrent/Metastatic Human Papilloma Virus Associated Cancers*. (NCT03439085) [accessed on 09.12.2025]; Available from: https://clinicaltrials.gov/study/NCT03439085.

[60] Ottensmeier, C. H. H. et al. 999MO HARE-40: A phase I/II trial of therapeutic HPV vaccine (BNT113) in patients with HPV16 driven carcinoma. *Ann. Oncol.* **35**, S680 (2024).

[61] Saba, N. F. et al. Exploratory efficacy and translational results from the safety run in of AHEAD-MERIT, a phase II trial of first line pembrolizumab plus the fixed-antigen cancer vaccine BNT113 in advanced HPV16+ HNSCC. *Ann. Oncol.* **35**, S627 (2024).

[62] Even, C. et al. Preliminary results of a multicentric randomized phase I/IIa trial of an immunotherapy targeting dendritic cells (DC), CD40HVac, in patients with HPV16-positive oropharyngeal carcinoma (OPC). *IOTECH* **24** (2024).

[63] Weiss, J. et al. VERSATILE-002: Survival with first-line treatment with PDS0101 therapeutic vaccine and pembrolizumab in HPV16-positive recurrent/metastatic head and neck squamous cell carcinoma (HNSCC). *Ann. Oncol.* **35**, S628 (2024).

[64] Routman, D. M. et al. Initial results of MC200710 investigating therapeutic vaccine (PDS0101) alone or with pembrolizumab prior to surgery or radiation therapy for locally advanced HPV associated oropharyngeal carcinoma, a phase 2 window of opportunity trial. *J. Clin. Oncol.* **43**, 6061–6061 (2025).

[65] Grippin, A. et al. IMMUNOCERV phase II trial combining the HPV-specific T cell immunotherapy PDS0101 with chemoradiation for treatment of locally advanced cervical cancer. *Int. J. Radiat. Oncol. Biol. Phys.* **120**, S113 (2024).

[66] Melief, C. J. M. et al. Strong vaccine responses during chemotherapy are associated with prolonged cancer survival. *Sci. Transl. Med.* **12**, eaaz8235 (2020). 32188726 10.1126/scitranslmed.aaz8235

[67] Massarelli, E. et al. Combining immune checkpoint blockade and tumor-specific vaccine for patients with incurable human papillomavirus 16-related cancer: a phase 2 clinical trial. *JAMA Oncol* **5**, 67–73 (2019). 30267032 10.1001/jamaoncol.2018.4051 PMC6439768

[68] Sousa, L. G. et al. ISA101 and nivolumab for HPV-16(+) cancer: updated clinical efficacy and immune correlates of response. *JITC* **10** (2022). 10.1136/jitc-2021-004232 PMC9066369 35193933

[69] Even, C. et al. Randomized clinical efficacy and safety study of peltopepimut-S plus cemiplimab compared to cemiplimab alone in patients with recurrent/metastatic HPV16-positive head and neck cancer. *JITC* **13**, e012555 (2025). 40921630 10.1136/jitc-2025-012555 PMC12421166

[70] Lorusso, D. et al. Cemiplimab plus peltopepimut-S vaccine in recurrent cervical cancer: A phase 2 clinical trial. *Gynecol. Oncol.* **196**, 28–35 (2025). 40154184 10.1016/j.ygyno.2025.03.019

[71] Colevas, A. D. et al. A phase 1 dose-escalation and expansion study of CUE-101, given as monotherapy and in combination with pembrolizumab, in patients with recurrent/metastatic HPV16+ head and neck squamous cell cancer (R/M HNSCC). *J. Clin. Oncol.* **42**, 6004–6004 (2024).

[72] Puram, S. et al. Safety of administration of CUE-101, a novel HPV16 E7-pHLA-IL2-Fc fusion protein, before curative-intent treatment in patients with HPV16+ oropharyngeal squamous cell carcinoma. *J. Clin. Oncol.* **43**, e18086–e18086 (2025).

[73] Nakagawa, M. et al. A randomized double-blind phase 2 clinical trial treating cervical intraepithelial neoplasia 2/3 with PepCan or Candida. *medRxiv*, 2025.2001.2018.25320725 (2025).

[74] University of Arkansas. *A Phase I/II Clinical Trial of PepCan in Head and Neck Cancer Patients in Remission to Reduce Recurrence Regardless of HPV Status*. (NCT03821272) [accessed on 09.12.2025]; Available from: https://clinicaltrials.gov/study/NCT03821272.

[75] Borcoman, E. et al. Phase Ib/II trial of tipapkinogene sovacivec, a therapeutic human papillomavirus16-vaccine, in combination with avelumab in patients with advanced human papillomavirus16-positive cancers. *Eur. J. Cancer* **191**, 112981 (2023). 37506588 10.1016/j.ejca.2023.112981

[76] Le Tourneau, C. et al. Randomized phase II trial evaluating the combination of TG4001, an HPV16 therapeutic vaccine, and avelumab (ave) in patients (pts) with immunotherapy-naïve recurrent and/or metastatic (R/M) HPV16-positive cervical or anogenital cancer. *J. Clin. Oncol.* **43**, 2638–2638 (2025).

[77] Floudas, C. S. et al. PRGN-2009 and bintrafusp alfa for patients with advanced or metastatic human papillomavirus-associated cancer. *Cancer Immunol. Immunother.* **74**, 155 (2025). 40116923 10.1007/s00262-025-04009-z PMC11928712

[78] Bracken-Clarke, D. M. et al. Phase II trial of immunotherapeutic HPV vaccine PRGN-2009 with pembrolizumab before standard treatment in subjects with newly diagnosed HPV-associated oropharyngeal cancer. *J. Clin. Oncol.* **42**, TPS6124–TPS6124 (2024).

[79] Floudas, C. S. et al. Phase II trial of neoadjuvant chemotherapy (NAC) docetaxel-cisplatin alone (DC) or with anti-human papillomavirus (HPV) gene therapy PRGN-2009 (DCP) followed by surgery in patients (pts) with newly diagnosed HPV-associated oropharyngeal cancer (HPV-OPC). *J. Clin. Oncol.* **43**, TPS6117–TPS6117 (2025).

[80] Tschernia, N. et al. A phase 2 study to evaluate efficacy and safety of PRGN-2009, a novel gorilla adenovirus-based immunotherapy, in combination with pembrolizumab versus pembrolizumab monotherapy in patients with recurrent or metastatic cervical cancer. *J. Clin. Oncol.* **42**, TPS5623–TPS5623 (2024).

[81] Bakker, N. A. M. et al. HPV-16 E6/E7 DNA tattoo vaccination using genetically optimized vaccines elicit clinical and immunological responses in patients with usual vulvar intraepithelial neoplasia (uVIN): a phase I/II clinical trial. *JITC* **9** (2021). 10.1136/jitc-2021-002547 PMC8330588 34341131

[82] Palefsky, J. M. et al. A trial of SGN-00101 (HspE7) to treat high-grade anal intraepithelial neoplasia in HIV-positive individuals. *AIDS* **20**, 1151–1155 (2006). 16691066 10.1097/01.aids.0000226955.02719.26

[83] Einstein, M. H. et al. Heat shock fusion protein-based immunotherapy for treatment of cervical intraepithelial neoplasia III. *Gynecol. Oncol.* **106**, 453–460 (2007). 17586030 10.1016/j.ygyno.2007.04.038 PMC2013935

[84] Van Damme, P. et al. GTL001, a therapeutic vaccine for women infected with human papillomavirus 16 or 18 and normal cervical cytology: Results of a Phase I clinical trial. *Clin. Cancer Res.* **22**, 3238–3248 (2016). 27252412 10.1158/1078-0432.CCR-16-0085

[85] Sun, H. X., Xie, Y. & Ye, Y. P. ISCOMs and ISCOMATRIX. *Vaccine* **27**, 4388–4401 (2009). 19450632 10.1016/j.vaccine.2009.05.032

[86] Anderson, J. S. et al. A randomized, placebo-controlled, dose-escalation study to determine the safety, tolerability, and immunogenicity of an HPV-16 therapeutic vaccine in HIV-positive participants with oncogenic HPV infection of the anus. *JAIDS J. Acquired Immune Defic. Syndromes* **52** (2009). 10.1097/QAI.0b013e3181b7354c 19661810

[87] Frazer, I. H. et al. Phase 1 study of HPV16-specific immunotherapy with E6E7 fusion protein and ISCOMATRIX™ adjuvant in women with cervical intraepithelial neoplasia. *Vaccine* **23**, 172–181 (2004). 15531034 10.1016/j.vaccine.2004.05.013

[88] Hallez, S. et al. Phase I/II trial of immunogenicity of a human papillomavirus (HPV) type 16 E7 protein–based vaccine in women with oncogenic HPV-positive cervical intraepithelial neoplasia. *Cancer Immunol. Immunother.* **53**, 642–650 (2004). 14985860 10.1007/s00262-004-0501-4 PMC11034211

[89] Kaufmann, A. M. et al. Vaccination trial with HPV16 L1E7 chimeric virus-like particles in women suffering from high grade cervical intraepithelial neoplasia (CIN 2/3). *Int. J. Cancer* **121**, 2794–2800 (2007). 17721997 10.1002/ijc.23022

[90] Speetjens, F. M. et al. Intradermal vaccination of HPV-16 E6 synthetic peptides conjugated to an optimized Toll-like receptor 2 ligand shows safety and potent T cell immunogenicity in patients with HPV-16 positive (pre-)malignant lesions. *JITC* **10** (2022). 10.1136/jitc-2022-005016 PMC9582304 36261215

[91] Solares, A. M. et al. Safety and immunogenicity of a human papillomavirus peptide vaccine (CIGB-228) in women with high-grade cervical intraepithelial neoplasia: first-in-human, proof-of-concept trial. *ISRN Obstet. Gynecol.* **2011**, 292951 (2011). 21748025 10.5402/2011/292951 PMC3118643

[92] Dana-Farber Cancer Institute. *A Phase Ib/II Trial To Test The Safety And Efficacy Of Vaccination With HPV16-E711-19 Nanomer For The Treatment Of Incurable HPV 16-Related Oropharyngeal, Cervical And Anal Cancer In HLA-A*02 Positive Patients*. (NCT02865135) [accessed on 09.12.2025]; Available from: https://clinicaltrials.gov/study/NCT02865135.

[93] Otterhaug, T. et al. Photochemical internalization enhanced vaccination is safe, and gives promising cellular immune responses to an HPV peptide-based vaccine in a phase I clinical study in healthy volunteers. *Front. Immunol.* **11**, 576756 (2020). 33488576 10.3389/fimmu.2020.576756 PMC7819858

[94] Choi, C. H. et al. Efficacy and safety of BVAC-C in HPV type 16- or 18-positive cervical carcinoma who failed 1st platinum-based chemotherapy: a phase I/IIa study. *Front. Immunol.* **15**, 1371353 (2024). 38605958 10.3389/fimmu.2024.1371353 PMC11007103

[95] Ferrara, A. et al. Dendritic cell-based tumor vaccine for cervical cancer II: results of a clinical pilot study in 15 individual patients. *J. Cancer Res. Clin. Oncol.* **129**, 521–530 (2003). 12898233 10.1007/s00432-003-0463-5 PMC12161992

[96] Santin, A. D. et al. HPV16/18 E7-pulsed dendritic cell vaccination in cervical cancer patients with recurrent disease refractory to standard treatment modalities. *Gynecol. Oncol.* **100**, 469–478 (2006). 16249018 10.1016/j.ygyno.2005.09.040

[97] Santin, A. D. et al. Human papillomavirus type 16 and 18 E7-pulsed dendritic cell vaccination of stage IB or IIA cervical cancer patients: a phase I escalating-dose trial. *J. Virol.* **82**, 1968–1979 (2008). 18057249 10.1128/JVI.02343-07 PMC2258728

[98] Ho, A. L. et al. HB-200 arenavirus-based immunotherapy plus pembrolizumab as first-line treatment of patients with recurrent/metastatic HPV16-positive head and neck cancer: Updated results. *J. Clin. Oncol.* **42**, 6005–6005 (2024).

[99] García-Hernández, E. et al. Regression of papilloma high-grade lesions (CIN 2 and CIN 3) is stimulated by therapeutic vaccination with MVA E2 recombinant vaccine. *Cancer Gene Ther* **13**, 592–597 (2006). 16456551 10.1038/sj.cgt.7700937

[100] Albarran, Y. C. A. et al. MVA E2 recombinant vaccine in the treatment of human papillomavirus infection in men presenting intraurethral flat condyloma: a phase I/II study. *Biodrugs* **21**, 47–59 (2007). 17263589 10.2165/00063030-200721010-00006

[101] Huh, W. K. et al. Phase II study of axalimogene filolisbac (ADXS-HPV) for platinum-refractory cervical carcinoma: An NRG oncology/gynecologic oncology group study. *Gynecol. Oncol.* **158**, 562–569 (2020). 32641240 10.1016/j.ygyno.2020.06.493 PMC7487015

[102] Park, Y. C. et al. A phase 1/2a, dose-escalation, safety and preliminary efficacy study of oral therapeutic vaccine in subjects with cervical intraepithelial neoplasia 3. *J. Gynecol. Oncol.* **30**, e88 (2019). 31576684 10.3802/jgo.2019.30.e88 PMC6779607

[103] Kawana, K. et al. Oral vaccination against HPV E7 for treatment of cervical intraepithelial neoplasia grade 3 (CIN3) elicits E7-specific mucosal immunity in the cervix of CIN3 patients. *Vaccine* **32**, 6233–6239 (2014). 25258102 10.1016/j.vaccine.2014.09.020

[104] Santos, C., Vilanova, M., Medeiros, R. & Gil da Costa, R. M. HPV-transgenic mouse models: Tools for studying the cancer-associated immune response. *Virus Res* **235**, 49–57 (2017). 28385491 10.1016/j.virusres.2017.04.001

[105] Rösl, F., Schäfer, K. & Nafz, J. Mastomys Coucha: a natural animal model for papillomavirus-induced skin carcinogenesis. *Cent. Eur. J. Public Health* **16** (2008).

[106] Ingle, A. et al. Novel laboratory mouse papillomavirus (MusPV) infection. *Vet. Pathol.* **48**, 500–505 (2011). 20685915 10.1177/0300985810377186

[107] Hu, J., Cladel, N. M., Budgeon, L. R., Balogh, K. K. & Christensen, N. D. The mouse papillomavirus infection model. *Viruses* **9**, 246 (2017). 28867783 10.3390/v9090246 PMC5618012

[108] Schulz, E. et al. Isolation of three novel rat and mouse papillomaviruses and their genomic characterization. *PLoS One* **7**, e47164 (2012). 23077564 10.1371/journal.pone.0047164 PMC3471917

[109] Vinzón, S. E. et al. Protective vaccination against papillomavirus-induced skin tumors under immunocompetent and immunosuppressive conditions: a preclinical study using a natural outbred animal model. *PLoS Pathog* **10**, e1003924 (2014). 24586150 10.1371/journal.ppat.1003924 PMC3930562

[110] Mao, M. et al. Immunological dynamics in orthotopic versus subcutaneous murine models of HPV-positive oropharyngeal cancer. *Dis. Model. Mech*., dmm.052311 (2025). 10.1242/dmm.052311 PMC12746710 40899264

[111] Lin, K. Y. et al. Treatment of established tumors with a novel vaccine that enhances major histocompatibility class II presentation of tumor antigen. *Cancer Res.* **56**, 21–26 (1996). 8548765

[112] Decrausaz, L. et al. A novel mucosal orthotopic murine model of human papillomavirus-associated genital cancers. *Int. J. Cancer* **128**, 2105–2113 (2011). 20635385 10.1002/ijc.25561

[113] Sandoval, F. et al. Mucosal imprinting of vaccine-induced CD8+ T cells is crucial to inhibit the growth of mucosal tumors. *Sci. Transl. Med.* **5**, 172ra120–172ra120 (2013). 10.1126/scitranslmed.3004888 PMC4086646 23408053

[114] Venuti, A., Curzio, G., Mariani, L. & Paolini, F. Immunotherapy of HPV-associated cancer: DNA/plant-derived vaccines and new orthotopic mouse models. *Cancer Immunol. Immunother.* **64**, 1329–1338 (2015). 26138695 10.1007/s00262-015-1734-0 PMC4554738

[115] Feltkamp, M. C. W. et al. Vaccination with cytotoxic T lymphocyte epitope-containing peptide protects against a tumor induced by human papillomavirus type 16-transformed cells. *Eur. J. Immunol.* **23**, 2242–2249 (1993). 7690326 10.1002/eji.1830230929

[116] Peng, S. et al. Development of a spontaneous HPV16 E6/E7-expressing head and neck squamous cell carcinoma in HLA-A2 transgenic mice. *mBio* **13**, e0325221 (2022). 35089069 10.1128/mbio.03252-21 PMC8725581

[117] Pajot, A. et al. A mouse model of human adaptive immune functions: HLA-A2.1-/HLA-DR1-transgenic H-2 class I-/class II-knockout mice. *Eur. J. Immunol.* **34**, 3060–3069 (2004). 15468058 10.1002/eji.200425463

[118] Dhamodaran, A. et al. Mice with a diverse human T cell receptor repertoire selected on multiple HLA class I molecules. *Nat. Commun.* **16**, 5432 (2025). 40593800 10.1038/s41467-025-61306-y PMC12216589

[119] Kruse, S. et al. Therapeutic vaccination using minimal HPV16 epitopes in a novel MHC-humanized murine HPV tumor model. *Oncoimmunology* **8**, e1524694 (2019). 30546964 10.1080/2162402X.2018.1524694 PMC6287800

[120] Zottnick, S., Voß, A. L. & Riemer, A. B. Inducing immunity where it matters: Orthotopic HPV tumor models and therapeutic vaccinations. *Front. Immunol.* **11**, 1750 (2020). 32922389 10.3389/fimmu.2020.01750 PMC7457000

[121] Schifflers, C. et al. Development of an orthotopic HPV16-dependent base of tongue tumor model in MHC-humanized mice. *Pathogens* **12** (2023). 10.3390/pathogens12020188 PMC9958775 36839460

[122] Ramos da Silva, J. et al. Single immunizations of self-amplifying or non-replicating mRNA-LNP vaccines control HPV-associated tumors in mice. *Sci. Transl. Med.* **15**, eabn3464 (2023). 36867683 10.1126/scitranslmed.abn3464

[123] Lee, S. et al. mRNA-HPV vaccine encoding E6 and E7 improves therapeutic potential for HPV-mediated cancers via subcutaneous immunization. *J. Med. Virol.* **95**, e29309 (2023). 38100632 10.1002/jmv.29309

[124] Li, S. et al. Local mucosal immunization of self-assembled nanofibers elicits robust antitumor effects in an orthotopic model of mouse genital tumors. *Nanoscale* **12**, 3076–3089 (2020). 31965136 10.1039/c9nr10334a

[125] Arribillaga, L. et al. Bivalent therapeutic vaccine against HPV16/18 genotypes consisting of a fusion protein between the extra domain A from human fibronectin and HPV16/18 E7 viral antigens. *JITC* **8** (2020). 10.1136/jitc-2020-000704 PMC7319778 32581060

[126] Silva, M. O. et al. Antigen delivery to DEC205(+) dendritic cells induces immunological memory and protective therapeutic effects against HPV-associated tumors at different anatomical sites. *Int. J. Biol. Sci.* **17**, 2944–2956 (2021). 34345218 10.7150/ijbs.57038 PMC8326119

[127] Nakahashi-Ouchida, R. et al. Cationic nanogel-based nasal therapeutic HPV vaccine prevents the development of cervical cancer. *Sci. Transl. Med.* **17**, eado8840 (2025). 41223246 10.1126/scitranslmed.ado8840

[128] Kruse, S. et al. A versatile silica nanoparticle platform for induction of T cell responses – applied for therapeutic vaccination against HPV16 E6/E7-positive tumors in MHC-humanized mice. *Oncoimmunology* **14**, 2548002 (2025). 40981650 10.1080/2162402X.2025.2548002 PMC12382477

[129] Zhang, Q. et al. mRNA-encoded mutant HPV16/18 vaccines promote specific T-cell responses and synergize with anti-PD-1 checkpoint blockade in mediating therapeutic tumor regression in mice. *JITC* **13**, e012090 (2025). 40967673 10.1136/jitc-2025-012090 PMC12458754

[130] Peng, S. et al. Therapeutic mRNA vaccines targeting human papillomavirus type 16 E7 protein demonstrate significant antitumor efficacy in murine models of HPV-associated tumors. *Int. J. Pharm.* **680**, 125785 (2025). 40446873 10.1016/j.ijpharm.2025.125785

[131] Ye, T. et al. CO-DELIVERY of glutamic acid-extended peptide antigen and imidazoquinoline TLR7/8 agonist via ionizable lipid nanoparticles induces protective anti-tumor immunity. *Biomaterials* **311**, 122693 (2024). 38996672 10.1016/j.biomaterials.2024.122693

[132] Zhang, K. et al. Heterologous prime-boost with an mRNA vaccine and an oncolytic virus enhances tumor regression through overcoming intratumoral immune suppression. *Cell Rep* **44**, 115745 (2025). 40411783 10.1016/j.celrep.2025.115745

[133] Kayyal, M., Bolhassani, A., Noormohammadi, Z. & Sadeghizadeh, M. In silico design and immunological studies of two novel multiepitope DNA-based vaccine candidates against high-risk human papillomaviruses. *Mol. Biotechnol.* **63**, 1192–1222 (2021). 34308516 10.1007/s12033-021-00374-z

[134] Zhu, L. et al. Design and evaluation of a multi-epitope DNA vaccine against HPV16. *Hum. Vaccin. Immunother.* **20**, 2352908 (2024). 38780076 10.1080/21645515.2024.2352908 PMC11123455

[135] Ehsasatvatan, M. & Baghban Kohnehrouz, B. Designing and immunomolecular analysis of a new broad-spectrum multiepitope vaccine against divergent human papillomavirus types. *PLoS One* **19**, e0311351 (2024). 39621646 10.1371/journal.pone.0311351 PMC11611089

[136] Ryan, N. et al. Immunoinformatics approach for design novel multi-epitope prophylactic and therapeutic vaccine based on capsid proteins L1 and L2 and oncoproteins E6 and E7 of human papillomavirus 16 and human papillomavirus 18 against cervical cancer. *PHRP* **15**, 307–328 (2024). 39039819 10.24171/j.phrp.2024.0013 PMC11391375

[137] Abramson, J. et al. Accurate structure prediction of biomolecular interactions with AlphaFold 3. *Nature* **630**, 493–500 (2024). 38718835 10.1038/s41586-024-07487-w PMC11168924

[138] Najafi, A., Ataee, M. H., Farzanehpour, M. & Esmaeili Guvarchin Ghaleh, H. A comparative analysis of computational strategies in multi-epitope vaccine design against human papillomavirus and cervical cancer. *Cell J* **26**, 403–426 (2024). 39290119 10.22074/cellj.2024.2028622.1568

[139] Olawade, D. B. et al. Leveraging artificial intelligence in vaccine development: a narrative review. *J. Microbiol. Methods* **224**, 106998 (2024). 39019262 10.1016/j.mimet.2024.106998

[140] Ibrahim Khalil, A., Zhang, L., Muwonge, R., Sauvaget, C. & Basu, P. Efficacy and safety of therapeutic HPV vaccines to treat CIN 2/CIN 3 lesions: a systematic review and meta-analysis of phase II/III clinical trials. *BMJ Open* **13**, e069616 (2023). 37879679 10.1136/bmjopen-2022-069616 PMC10603536

[141] Alouini, S. & Pichon, C. Therapeutic vaccines for HPV-associated cervical malignancies: a systematic review. *Vaccines* **12** (2024). 10.3390/vaccines12040428 PMC11054545 38675811

[142] Harper, D. M. et al. The efficacy and safety of Tipapkinogen Sovacivec therapeutic HPV vaccine in cervical intraepithelial neoplasia grades 2 and 3: randomized controlled phase II trial with 2.5 years of follow-up. *Gynecol. Oncol.* **153**, 521–529 (2019). 30955915 10.1016/j.ygyno.2019.03.250

[143] Choi, Y. J. et al. A phase II, prospective, randomized, multicenter, open-label study of GX-188E, an HPV DNA vaccine, in patients with cervical intraepithelial neoplasia 3. *Clin. Cancer Res.* **26**, 1616–1623 (2020). 31727676 10.1158/1078-0432.CCR-19-1513

[144] Ramanathan, P., Ganeshrajah, S., Raghanvan, R. K., Singh, S. S. & Thangarajan, R. Development and clinical evaluation of dendritic cell vaccines for HPV related cervical cancer-a feasibility study. *Asian Pac. J. Cancer Prev.* **15**, 5909–5916 (2014). 25081721 10.7314/apjcp.2014.15.14.5909

[145] Basu, P. et al. A randomized phase 2 study of ADXS11-001 Listeria monocytogenes-Listeriolysin O immunotherapy with or without cisplatin in treatment of advanced cervical cancer. *Int. J. Gynecol. Cancer* **28**, 764–772 (2018). 29538258 10.1097/IGC.0000000000001235 PMC5929492

[146] Wentzensen, N. et al. Clinical evaluation of human papillomavirus screening with p16/Ki-67 dual stain triage in a large organized cervical cancer screening program. *JAMA Intern. Med.* **179**, 881–888 (2019). 31081870 10.1001/jamainternmed.2019.0306 PMC6515572

[147] Trimble, C. L. et al. A phase I trial of a human papillomavirus DNA vaccine for HPV16+ cervical intraepithelial neoplasia 2/3. *Clin. Cancer Res.* **15**, 361–367 (2009). 19118066 10.1158/1078-0432.CCR-08-1725 PMC2865676

[148] Floudas, C. S. et al. Novel combination immunotherapy and clinical activity in patients with HPV-associated cancers: a nonrandomized clinical trial. *JAMA Oncol* **11**, 394–399 (2025). 39976981 10.1001/jamaoncol.2024.6998 PMC11843463

